# A unique structural domain in *Methanococcoides burtonii* ribulose-1,5-bisphosphate carboxylase/oxygenase (Rubisco) acts as a small subunit mimic

**DOI:** 10.1074/jbc.M116.767145

**Published:** 2017-01-30

**Authors:** Laura H. Gunn, Karin Valegård, Inger Andersson

**Affiliations:** From the Department of Cell and Molecular Biology, Uppsala University, S-751 24 Uppsala, Sweden

**Keywords:** archaea, carbon fixation, metal ion-protein interaction, oligomerization, protein evolution, ribulose-1,5-bisphosphate carboxylase/oxygenase (Rubisco), structure-function, X-ray crystallography

## Abstract

The catalytic inefficiencies of the CO_2_-fixing enzyme ribulose-1,5-bisphosphate carboxylase/oxygenase (Rubisco) often limit plant productivity. Strategies to engineer more efficient plant Rubiscos have been hampered by evolutionary constraints, prompting interest in Rubisco isoforms from non-photosynthetic organisms. The methanogenic archaeon *Methanococcoides burtonii* contains a Rubisco isoform that functions to scavenge the ribulose-1,5-bisphosphate (RuBP) by-product of purine/pyrimidine metabolism. The crystal structure of *M. burtonii* Rubisco (MbR) presented here at 2.6 Å resolution is composed of catalytic large subunits (LSu) assembled into pentamers of dimers, (L_2_)_5_, and differs from Rubiscos from higher plants where LSus are glued together by small subunits (SSu) into hexadecameric L_8_S_8_ enzymes. MbR contains a unique 29-amino acid insertion near the C terminus, which folds as a separate domain in the structure. This domain, which is visualized for the first time in this study, is located in a similar position to SSus in L_8_S_8_ enzymes between LSus of adjacent L_2_ dimers, where negatively charged residues coordinate around a Mg^2+^ ion in a fashion that suggests this domain may be important for the assembly process. The Rubisco assembly domain is thus an inbuilt SSu mimic that concentrates L_2_ dimers. MbR assembly is ligand-stimulated, and we show that only 6-carbon molecules with a particular stereochemistry at the C_3_ carbon can induce oligomerization. Based on MbR structure, subunit arrangement, sequence, phylogenetic distribution, and function, MbR and a subset of Rubiscos from the Methanosarcinales order are proposed to belong to a new Rubisco subgroup, named form IIIB.

## Introduction

Ribulose-1,5-bisphosphate (RuBP)^2^ carboxylase/oxygenase (Rubisco) catalyzes the carboxylation of RuBP to yield two molecules of 3-phosphoglycerate (3-PGA). In the majority of organisms harboring Rubisco, this is the initial and rate-limiting reaction in the reductive pentose phosphate cycle of photosynthesis leading to the incorporation of CO_2_ into the biosphere. Rubisco exhibits a slow CO_2_ fixation rate and poorly discriminates between substrate CO_2_ and O_2_. Fixation of O_2_ produces the toxic compound 2-phosphoglycolate whose recycling consumes energy and releases fixed CO_2_. Discerning between CO_2_ and O_2_ at the molecular level is fundamentally a difficult task and was unnecessary for ancestral Rubisco that arose under relatively sparse atmospheric O_2_ concentrations. Rubisco's catalytic inefficiencies often limit plant growth and resource-use-efficiency (1, 2). However, strategies to engineer more efficient plant Rubiscos have been thwarted by an inherent catalytic trade-off between turnover and specificity suggesting that higher plant Rubisco catalysis may have reached an evolutionary maximum (3). Catalytic improvements may be further complicated by a heavy reliance on co-evolved molecular partners (4) and an inability using current computational approaches to predict the assembly capacity and catalytic performance resulting from alterations in sequence and 3D structure (5). However, different Rubisco lineages have faced different selection pressures, particularly Rubisco isoforms from non-photosynthetic organisms (6). Understanding Rubisco's evolutionary history may free us from the bias of the evolutionary constraints placed upon higher plant Rubisco isoforms when developing and designing alternative CO_2_-fixing enzymes.

Catalysis in Rubisco occurs at the interface of two 50–52-kDa large subunits (LSu). This L_2_ dimer is the functional unit and contains two active sites. Form II (bacterial) and form III (archaeal) Rubiscos exist as such L_2_ dimers or as oligomers of dimers, (L_2_)*_n_*. In form I (higher plant, cyanobacterial, algal, and most proteobacterial) Rubiscos, four L_2_ dimers are assembled around a 4-fold axis by two tetramers of ∼15-kDa small subunits (SSu) that cap each end of the (L_2_)_4_ barrel. These ∼550-kDa hexadecameric L_8_S_8_ Rubiscos exhibit far superior catalytic efficiency than their (L_2_)*_n_* counterparts. The SSu is thought to have evolved before the divergence of proteobacteria and cyanobacteria, but after the transfer of an archaeal form III Rubisco to eubacteria (7), and may share a common ancestor with a protein involved in Rubisco compartmentalization in cyanobacteria (8).

Form III Rubiscos do not contribute to photosynthetic processes *in vivo*, but because the catalytic mechanism is conserved, they can functionally substitute for photosynthetic Rubiscos (9), albeit with a low specificity factor and carboxylation rate (10). The Rubisco from the Antarctic-dwelling methanogenic archaeon *Methanococcoides burtonii* likely acts as part of the AMP/ADP-recycling pathway by scavenging the RuBP by-product (11, 12). *M. burtonii* Rubisco (MbR) has many intriguing characteristics. Five L_2_ dimers assemble into a rare (L_2_)_5_ structure observed exclusively in thermostable archaeal Rubiscos (13, 14). Unlike other (L_2_)_5_ enzymes, MbR oligomerization is substrate-stimulated (10). MbR contains a unique 26–30-amino acid insertion between α6 and β7 at the bottom of the βα-barrel (7, 10), and deleting this “bonus” sequence yields enzymes that are catalytically competent, albeit with altered kinetic properties, and are incapable of assembling into (L_2_)_5_ enzymes (15). MbR exhibits higher sequence homology to proteobacterial form II Rubiscos, and on this basis it has been classified as a form II Rubisco (7), despite being of archaeal origin and exhibiting archaeon-like decameric assembly.

A lack of structural information hinders MbR classification and prevents mechanistic insights into the role of the bonus sequence in substrate-induced oligomerization. Here, we describe the crystal structure of (L_2_)_5_ MbR, and by comparing MbR sequence and structure elements with other Rubisco lineages, we propose a re-evaluation of the current Rubisco classification system, establishing a new Rubisco group, form IIIB, which contains a subset of methanogenic archaeal Rubiscos with well defined sequence and phylogenetic characteristics. Biochemical approaches complement 3D structural analyses to describe the role of the unique bonus sequence as an inbuilt Rubisco SSu.

## Results

### Overall structure of MbR

**Figure 1.**
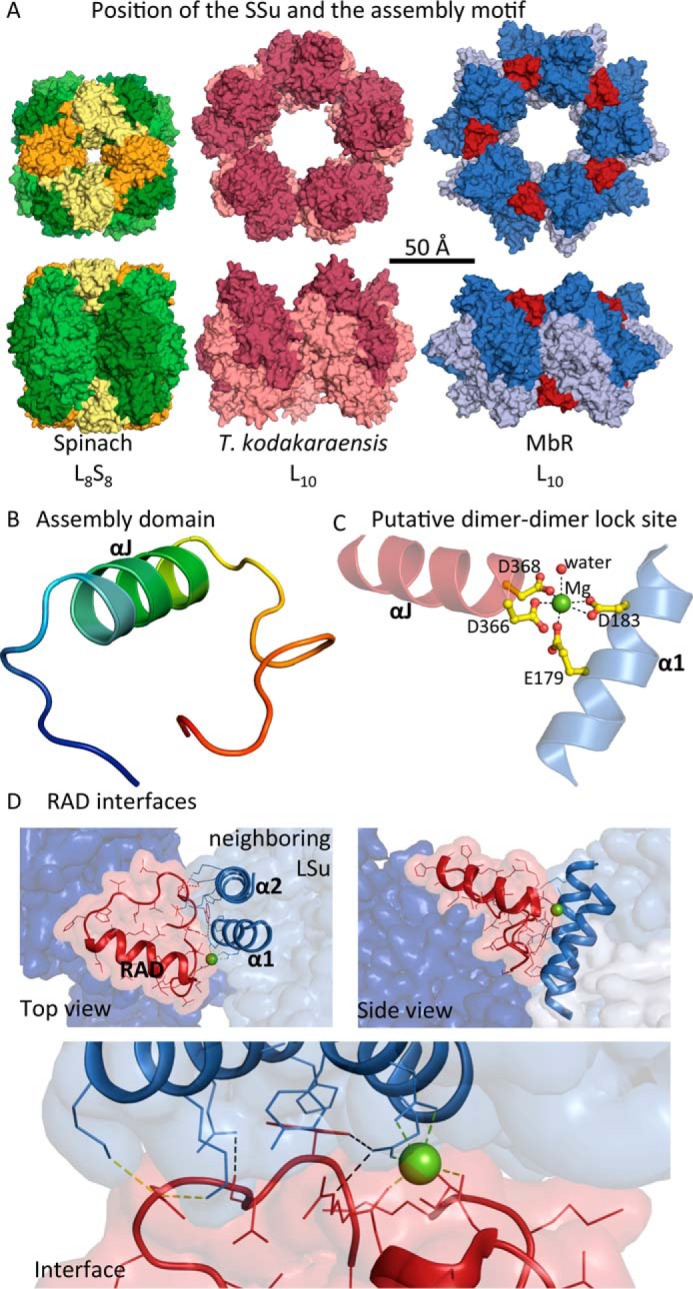
**MbR has its own inbuilt SSu that concentrates LSu dimers.** *A,* comparison of the position of the Rubisco SSu and MbR RAD in relation to Rubisco LSus: top and side views of the 3D crystal structures of *Spinacia oleracea* (spinach) form I L_8_S_8_ Rubisco (LSus and SSus *green* and *yellow*, respectively; PDB code 8RUC); *T. kodakarensis* form II L_10_ Rubisco (LSus, *pink*; PDB code 1GEH); MbR L_10_ Rubisco (LSus, *blue;* RAD, *red*). In the top MbR structure, five L_2_ dimers are arranged around a non-crystallographic 5-fold axis perpendicular to the page. *B,* structure of the MbR RAD rainbow-colored from *blue* at the C terminus to *red* at the N terminus. The RAD was defined from structural alignments as residues 361–389. *C,* potential locking mechanism between αJ of the RAD (*red*) and α1 of a neighboring Rubisco LSu (*blue*) involves the coordination of four negatively charged side chains and a solvent molecule around a magnesium ion. *D,* RAD (*red*) packs against its own LSu (*dark blue*) and interacts with a neighboring LSu (*light blue*). The interface is formed between loop residues in the RAD and residues in α1 and α2 of the neighboring LSu. Lock site, ionic, and hydrogen-bonding interactions at the interface are shown as *green, yellow,* and *black dashed lines*, respectively. The top and side views correspond to the views shown in *A*.

The MbR model presented here is essentially complete. Of the 474 residues, amino acids 1–473 could be modeled with the exception of residue 473 in chain C and residue 1 in chain E, which were not included in the model because of weak supporting electron density. The completeness of the MbR model is thus higher than previous structures of form II or form III Rubisco enzymes, including the *Rhodopseudomonas palustris* search model (Table 1). The MbR asymmetric unit contains five LSus. Applying the crystallographic symmetry results in the L_10_ biological unit (Fig. 1*A*) consistent with previous biochemical observations (10).

**Table 1:** **Comparison of available form II and form III Rubisco structures** The function and lineage of all proteobacterial form II and archaeal form III Rubiscos with 3D crystal structures are listed alongside an evaluation of their sequence and structural homology to MbR. Structural superpositions were performed using chain A from the PDB coordinates 5MAC (MbR), 5RUB (*R. rubrum*), 4LF1 (*R. palustris*), 5C2G (Gallionaceae sp.), 1GEH (*T. kodakarensis*), and 2D69 (*P. horikoshii*).

Rubisco form	Source organism	Quarternary structure	No. of amino acids per LSu	No. of amino acids per LSu in structure	Sequence similarity to MbR	Superposition with MbR LSu		Superposition with *R. rubrum* LSu	
						No. of aligned residues	r.m.s.d.	No. of aligned residues	r.m.s.d.
					%		Å		Å
Form II	*R. rubrum*	L_2_	490	436	39	351	1.40		
	*R. palustris*	L_6_	481	457	40	424	1.30	364	0.95
	Gallionellaceae sp.	L_6_	479	461	38	424	1.25	368	0.92
Form II (?)	*M. burtonii*	L_2_, L_4_ … L_10_	474	473				352	1.42
Form III	*T. kodakarensis*	L_10_	444	427	33	395	1.59	339	1.67
	*P. horikoshii*	L_8_	430	424	34	385	1.45	342	1.63

The overall MbR LSu structure possesses the characteristic Rubisco fold, consisting of an N-terminal domain and the C-terminal 8-stranded βα-barrel domain (see supplemental Figs. S1 and S2 for an explanation of the naming conventions for Rubisco secondary structure). The MbR structure (half of the biological unit) contains 10 Mg^2+^ ions, 5 Cl^−^ ions, and 294 water molecules. As expected, Mg^2+^ binds to the active site (5 per half-L_10_ decamer), stabilizing the carbamate formed at the active site lysine (16). A second binding site for Mg^2+^ was also inferred from difference electron density at the interface of the L_2_ dimers (two Mg^2+^ per interface and 5 per half-L_10_ decamer). This Mg^2+^ ion is coordinated by a structural motif unique to MbR, which adopts a loop-helix-loop conformation (Fig. 1*B*). The motif, which forms a separate domain, is encoded by a C-terminal 29-amino acid insertion in MbR (residues 361–389) and corresponds to a β-hairpin motif predicted from sequence data (17). The helix of the motif (named αJ) is located between helix α6 and strand β7 of the βα-barrel (supplemental Figs. S1 and S2). A search using the Dali server (18) did not identify structures that exhibit structural similarity to this new motif. Although the search model was at the lower size limit for the Dali server, these results suggest that the structure of this motif is unique. Because of its strategic location and its clear functional role (see below), we named it the Rubisco assembly domain (RAD). The Mg^2+^ ion is ligated by oxygen donor atoms provided by the side chains of Asp-366 and Asp-368 located at the N-terminal end of the RAD helix αJ (supplemental Fig. S1) and Glu-179 and Asp-183 located in helix α1 of the βα-barrel in the LSu of the neighboring L_2_ dimer (Fig. 1*C* and supplemental Fig. S1). A solvent molecule donates a further oxygen atom such that the lock site exhibits near-octahedral coordination geometry with a coordination number of 6 as expected for a magnesium ion (Fig. 1*C*) (19). Whereas the carboxylates of Glu-179 and Asp-368 are monodentate, the carboxylates of Asp-183 and Asp-366 tend toward bidentate in the equatorial plane; it is likely that at least one is bidentate at any one instant. The coordination of ligands to the Mg^2+^ ion from two adjacent subunits potentially distorts the coordination sphere, which may be considered somewhat between octahedral and trigonal bipyramidal (20). The minor deviations in geometry observed may be a result of the limited resolution or caused by crystal packing forces. However, more likely, the location at the interface between two LSus is the cause of the deviation from ideal magnesium coordination geometry. In addition to the Mg^2+^ lock site, the interaction surface between the RAD and the neighboring dimer is formed between residues 385 and 389 in the loop region of the RAD and residues in helices α1 and α2 in a neighboring LSu. This interface is likely stabilized by (i) salt bridges between Asp-385 and Lys-219 and Lys-222 in helix α2 and (ii) hydrogen bonding between the side chains of RAD residue Ser-387 and residue Glu-215 in helix α2. Aside from its role as a ligand to the lock site Mg^2+^ ion, residue Glu-179 is also engaged in hydrogen bonding to residues Trp-388 and Arg-389 of the RAD (Fig. 1*D*).

The modeling of solvent and metal ions at the current resolution (2.6 Å) is non-trivial. We also considered Na^+^ (20), which was present during crystallization, but favor Mg^2+^ on the grounds of geometry and chemistry. *M. burtonii* has a magnesium transporter, CorA, for active uptake of Mg^2+^ (21, 22), and it requires greater than 0.01 m MgSO_4_ or MgCl_2_ for growth.

### MbR assembly domain, an inbuilt SSu mimic

The MbR RAD is located between neighboring L_2_ dimers in a similar position as the SSu in L_8_S_8_ Rubiscos (Fig. 1*A*). This indicates that it may play a similar role to the SSu, concentrating LSu dimers. This type of concentrating structure is lacking in the archaeal *Thermococcus kodakarensis* L_10_ Rubisco (Fig. 1*A*).

Rubisco from *T. kodakarensis* is known to be more thermostable as a L_10_ decamer than a L_2_ dimer (14), but there is as yet no molecular explanation for this stability. There is no MbR-like Mg^2+^-facilitated lock site in *T. kodakarensis* Rubisco, and packing at the *T. kodakarensis* Rubisco dimer-dimer interface appears to be primarily stabilized by hydrogen bonding and salt bridges. In general, ionic interactions are insufficient to maintain the structure of thermostable proteins because the p*K_a_* values of positively charged residues are sensitive to temperature fluctuations (23). Therefore, the reason L_10_ *T. kodakarensis* Rubisco is so thermostable remains unclear. The optimal temperature for MbR catalysis is 55 °C (10), but it is also not known whether an L_10_ Rubisco arrangement is intrinsically more thermostable.

**Figure 2.**
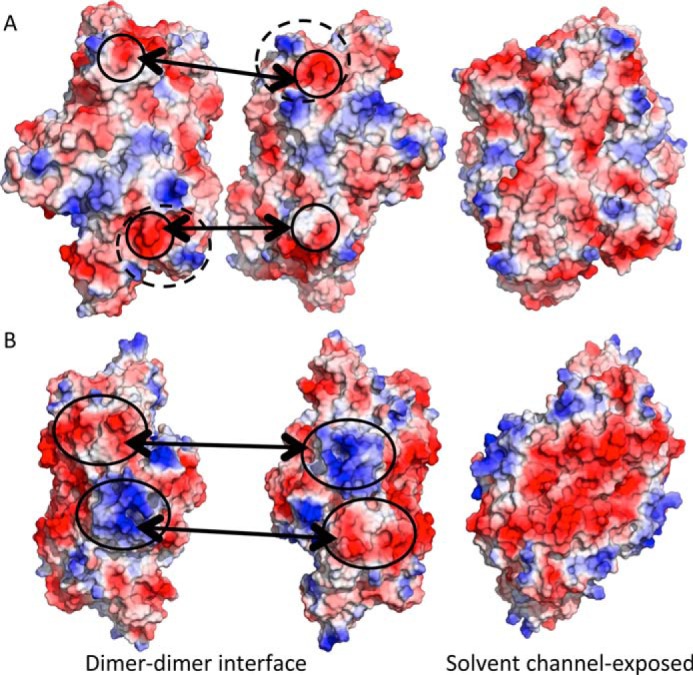
**Comparison of the electrostatic surface potential of L_2_ Rubisco dimers from *T. kodakarensis* and *M. burtonii*.** Electrostatic surface potential at the interface between L_2_ dimers and at the surface that lies within the Rubisco solvent channel in MbR (*A*) and *T. kodakarensis* (*B*). Electrostatic surfaces are colored *blue* in positive regions and *red* in negative regions. The regions corresponding to the MbR dimer-dimer lock site and the complementary charges at the *T. kodakarensis* Rubisco dimer-dimer interface are indicated by *solid circles*. The location of the RAD is indicated by *dashed circles* in *A*.

Analysis of the electrostatic surface charges of the MbR and *T. kodakarensis* L_2_ dimers indicates that the interface between L_2_ dimers (dimer-dimer interface) in *T. kodakarensis* includes a large number of charged residues positioned such that an extensive network of ionic interactions could facilitate dimer-dimer packing (Fig. 2*B*, *solid circles*). Although there are compatible charges at the MbR dimer-dimer interface (Fig. 2*A*), these are not as densely concentrated as in *T. kodakarensis* Rubisco, and the most highly charged areas are the negatively charged patches at the proposed lock site (Fig. 2*A*, *solid circles*). The solvent channel (interior) of L_10_ *T. kodakarensis* is highly negatively charged, which is not observed in the MbR structure (Fig. 2), and it may somehow contribute to *T. kodakarensis* thermostability. Furthermore, whereas salt bridges may act to stabilize the MbR L_10_ complex, the coordination of glutamic and aspartic acid residues (whose p*K_a_* are not drastically affected by temperature) to Mg^2+^ may represent the major locking mechanism of MbR L_2_ dimers, tethering them against one another to form higher oligomeric complexes.

### MbR oligomerization

**Figure 3.**
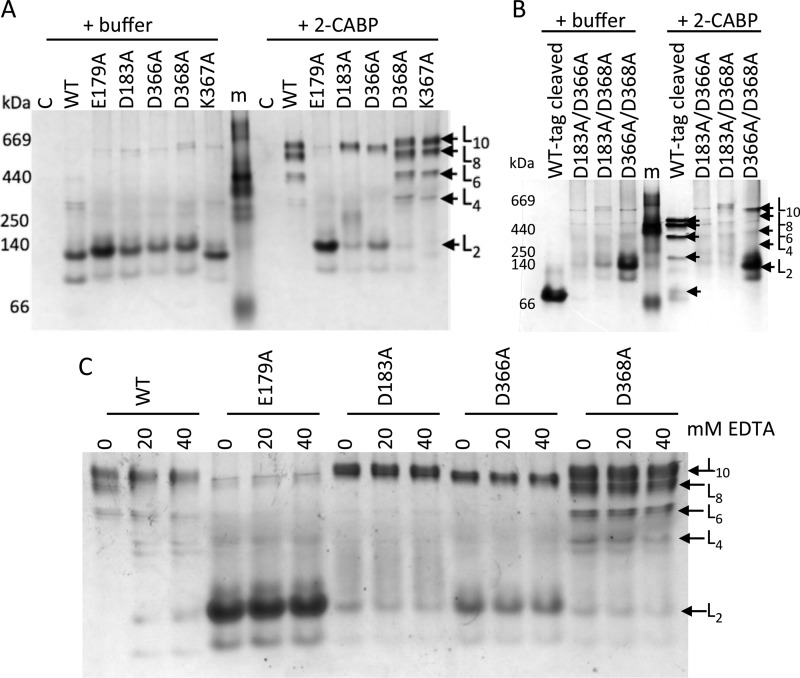
**Oligomerization potential of MbR harboring site-specific mutations.** Non-denaturing PAGE analyses of IMAC-purified single-MbR mutants (*A*) and double-MbR mutants (*B*) incubated with a 10× molar concentration of 2-CABP (relative to the number of Rubisco active sites). *C,* 2-CABP-bound wild-type MbR and putative lock site single mutants from *A* were incubated with increasing concentrations of EDTA. 5 μg of protein was loaded per lane. *Lane m*, protein molecular mass marker, sizes shown in kDa; *lane C*, pHUE empty-vector negative control; *lane WT*, wild-type MbR (positive control); *lane WT-tag cleaved,* wild-type MbR without a H_6_-Ub tag. Protein bands corresponding to distinct MbR oligomeric states are indicated.

Site-directed mutagenesis was undertaken to provide biochemical confirmation of the proposed α1-αJ lock site (Fig. 1*C*). The negatively charged residues Glu-179, Asp-183, Asp-366, and Asp-368, which appear to facilitate the RAD-locking mechanism, were mutated to alanine. The positively charged residue Lys-367, which is situated between the putative Asp-366 and Asp-368 lock residues, was also mutated to alanine to determine whether this residue is important for stabilizing the lock site. Higher order oligomer formation was only impeded by the E179A mutation (Fig. 3*A*). Residues Asp-183, Asp-366, and Asp-368 lie in an equatorial plane around the magnesium ion with the potential to contribute up to five bonds to the lock site (Fig. 1*C*), and thus disrupting any one of these three residues could be compensated for by the enzyme. In contrast, Glu-179 is the only axial protein ligand to Mg^2+^ (the other is provided by solvent), which could explain why the E179A mutation could disrupt the lock site and impede higher MbR oligomer formation (Fig. 1*C*). The long positively charged side chain of Lys-367 does not seem to be important for correct positioning of the neighboring Asp-366 and Asp-368.

Mutants E179A, D183A, D366A, and D368A MbR on the dimer-dimer interface exhibited altered migration on non-denaturing PAGE (Fig. 3*A*). In contrast, the K367A mutant migrated like unmodified MbR. Clearly, disrupting any one of the negative charges of the protein side chains at the lock site alters the charge to mass ratio and/or the hydrodynamic size of L_2_ MbR. However, no difference in L_2_ (or higher oligomer) migration was observed in the presence of 2-carboxy-arabinitol 1,5-bisphosphate (2-CABP). These data suggest that a structural rearrangement is induced in both the assembly domain and helix α1 upon substrate binding, even if the mutant enzyme is unable to oligomerize.

In the presence of 2-CABP, MbR with the D183A or D366A mutations was predominantly in the L_10_ form (Fig. 3*A*) rather than a mixture of oligomeric assemblies observed in wild-type MbR and the D368A mutant or predominantly L_2_ as observed in the E179A mutant. The destabilizing effect caused by mutating Asp-183 and Asp-366 (*i.e.* the lock site residues that tend toward bidentate) could thus be regarded as intermediate between that of the E179A and D368A mutations.

To evaluate how tightly the magnesium ion is bound at the dimer-dimer lock site in higher order MbR complexes, the 2-CABP-bound MbR and the single mutant variants (Fig. 3*A*) were incubated with increasing concentrations of EDTA. Non-denaturing PAGE analyses indicated that EDTA was unable to pull apart higher order MbR complexes, even in the presence of 2-fold molar concentrations of EDTA (relative to the Mg^2+^ concentration in the buffer used for Rubisco activation and 2-CABP binding; Fig. 3*C*). The inability of EDTA to chelate MbR-bound Mg^2+^ indicates that the complex formed between Glu-179, Asp-183, Asp-366, and Asp-368 and Mg^2+^ is stable. Similarly, EDTA cannot chelate Mg^2+^ bound to the carbamylated lysine at the Rubisco active site (24).

The double mutations D183A/D366A, D183A/D366A, and D366A/D368A were also introduced into MbR. All double mutants impeded oligomerization of MbR dimers (Fig. 3*B*), confirming the importance of negatively charged side chains at the lock site for MbR oligomerization.

### Substrate-induced oligomerization; the ligand is important

**Figure 4.**
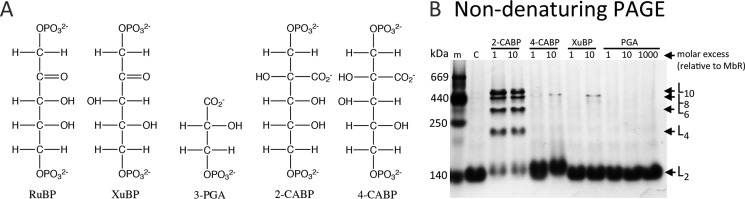
**Ligand binding to MbR.** *A,* ligands used in this study. *B,* non-denaturing PAGE protein separation. Purified and activated MbR were incubated with 1 or 10× molar concentrations of 2-CABP, 4-CABP, and XuBP and 1, 10, and 1000× molar concentrations of 3-PGA. 14 μg of protein was loaded per lane. *Lane m,* protein molecular mass marker, sizes shown (in kDa); *lane C,* purified MbR incubated with crystallization buffer.

It has been previously demonstrated that MbR oligomerization could be induced by substrate RuBP and the reaction-intermediate analog 2-CABP, whereas the Calvin-Benson-Bassham cycle intermediates 6-phosphogluconate, ribose 5-phosphate, fructose 6-phosphate, or fructose 1,6-bisphosphate failed to induce oligomerization (10). MbR was incubated with the ligands 4-carboxyarabinitol 1,5-bisphosphate (4-CABP), xylulose 1,5-bisphosphate (XuBP), and 3-PGA to further probe what triggers MbR oligomerization. 4-CABP and XuBP are the C3 epimers of 2-CABP and RuBP, respectively (Fig. 4*A*); 4-CABP is a close mimic of the reaction intermediate 2-carboxy-3-keto-d-arabinitol 1,5-bisphosphate, and XuBP is a misfire product of RuBP that is formed at carbamylated catalytic sites on Rubisco (25). Both XuBP and 4-CABP de-carbamylate the activator lysine in Rubisco during or after binding to the active site in the spinach enzyme (25, 26). Given their stereochemistry, the presence of these sugar compounds at the Rubisco active site precludes the presence of a metal ion (26). The product of Rubisco catalysis, 3-PGA, stabilizes loop 6 (supplemental Fig. S1) that folds over the active site during catalysis, but it does not induce complete closure of the loop (27).

Unlike 2-CABP, the ligands 4-CABP, XuBP, and 3-PGA did not induce MbR oligomerization above the small background level observed in negative controls (Fig. 4*B*). The inability of 10-fold molar concentrations (80 μm) of 4-CABP and XuBP to stimulate MbR oligomerization is striking, considering their similarity to the stereoisomers 2-CABP and RuBP, respectively, and their ability to bind to the active site of other Rubisco isoforms resulting in loop 6 closure (26). We conclude that only 6-carbon molecules that exhibit a particular stereochemistry at the C3 carbon induced MbR oligomerization (Fig. 4).

To examine differences between XuBP and 2-CABP binding to MbR, an MbR crystal structure was obtained to 2.8 Å resolution in the presence of XuBP (data not shown); however, poor data quality limited structural interpretation. Despite ligand binding assays indicating that L_2_ MbR does not oligomerize into higher order states in the presence of XuBP (Fig. 4*B*), the subunit arrangement in XuBP-incubated MbR crystals was L_10_. This L_10_ arrangement may be an artifact of crystallization. Just like the 2-CABP-bound MbR structure, the dimer-dimer lock site was held together by Mg^2+^ present in the crystallization buffer (data not shown). XuBP was in fact not bound, and the catalytic lysine was carbamylated. This suggests that either XuBP (i) cannot decarbamylate MbR or (ii) binds MbR so weakly that MbR was readily re-carbamylated by Mg^2+^ and NaHCO_3_ present in the crystallization buffer. Similarly, 4-CABP may not bind strongly, if at all, to MbR active sites.

Comparison of the 2-CABP-bound MbR structure with available Rubisco structures with XuBP and 4-CABP bound (PDB coordinates 1RCO and 1RBO of spinach Rubisco and 1RSC of *Synechococcus elongatus* Rubisco) revealed no dramatic differences in active site architecture, and the identity of catalytically significant residues is conserved in MbR but indicates that the stereochemistry at C3 of XuBP and 4-CABP may not be compatible with the geometry of the active site in the activated enzyme. Similar to the situation in Rubisco from higher plants, reversal of the configuration at C3 brings the C3 hydroxyl of the inhibitor closer to the position of the carbamoyl group of the activator lysine 193 (data not shown), but instead of decarbamylating the active site lysine, as observed in Rubisco from higher plants and cyanobacteria, MbR appears to expel XuBP from the active site. The reason for the different behavior in MbR is unclear, but it may be due to a low affinity of XuBP for MbR as was observed for form II and other non-higher plant Rubiscos. Future studies should determine the affinity of XuBP for the MbR active site.

A 1000-fold molar excess (8 mm) of 3-PGA was insufficient to induce MbR oligomerization (Fig. 4*B*). Assuming that MbR behaves the same in the presence of 3-PGA as other Rubisco isomers, this may indicate that loop 6 stabilization is not enough to induce oligomerization but that the complete closure of loop 6 is required. Given that only RuBP and 2-CABP can trigger MbR oligomerization, it seems likely that oligomerization is triggered by ligand binding at the active site and/or loop 6 closure.

### How may substrate binding affect the lock site?

**Figure 5.**
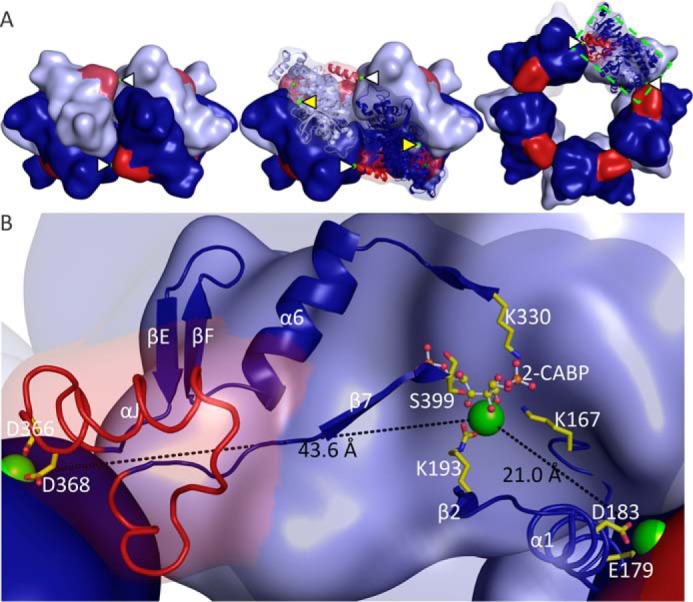
**Relative locations of the active and lock sites in MbR.** *A,* surface representations of L_10_ MbR. LSus are colored *dark/light blue,* and the assembly domain is highlighted in *red. Left* and *center,* side view of MbR showing lock sites at the interface between MbR dimers. *Right,* top view showing five lock sites. Lock and active sites are indicated by *white* and *yellow arrowheads*, respectively. *B,* close-up view of the *green boxed* region in *A, right,* showing the relative location of the lock sites (*sticks*) to the active site. Mg^2+^ ions are shown as *green spheres,* and 2-CABP bound at the active site is shown as *ball* and *sticks*. The helices αJ and α1 (*ribbon* representation) are linked to active site residues Lys-330, Ser-399, Lys-167, and Lys-193 (*sticks*) through defined structural elements (schematic representation). Distances between the active- and lock- site magnesium ions are indicated.

The RAD is linked to the catalytic site through helix α1 of the βα-barrel, which functions as a scaffold for the active site (Fig. 5). Loop 6 residue Lys-330, whose side chain can contact O7 or one of the carboxyl oxygens (O3P) of 2-CABP, is indirectly connected to the N terminus of the RAD via a 29-residue-long β-hairpin and α-helix segment. Similarly, residue Ser-399, whose main chain carbonyl contacts one of the P5 phosphate oxygens (O5P) of 2-CABP, connects to the C-terminal end of the RAD via a 9-amino acid extended structure. Residue Lys-167, located in the 8-amino acid long loop 1 of the βα-barrel that links to the N-terminal end of helix α1, binds 2-CABP at O1 and O6. The Mg^2+^ ion that stabilizes the carbamylated catalytic Lys-193 coordinates to O2, O3, and O6 of 2-CABP upon substrate binding. Lys-193 links via the 4-amino acid-long loop 2 to the C-terminal end of helix α1. The firm link of the active site to the RAD and/or α1 suggests that substrate binding could easily communicate some structural rearrangement to regions involved in the MbR lock site.

### Structural comparison with other Rubisco enzymes of bacterial and archaeal origin

The MbR structure was compared with available structures of form II and III Rubiscos as follows: the proteobacterial form II Rubiscos from *R. palustris*, *Rhodospirillum rubrum*, and Gallionellaceae sp., and archaeal form III Rubiscos from *T. kodakarensis* and *Pyrococcus horikoshii.* These form II and form III Rubisco structures exhibit low sequence similarity to MbR (33–40%; Table 1). *R. rubrum* functions as L_2_ dimers, whereas the *R. palustris* and Gallionaceae sp. Rubiscos form L_6_ hexamers. Unlike the form II Rubiscos, the archaeal form III *P. horikoshii* (L_8_) and *T. kodakarensis* (L_10_) Rubiscos function in non-photosynthetic pathways (Table 2).

**Table 2:**
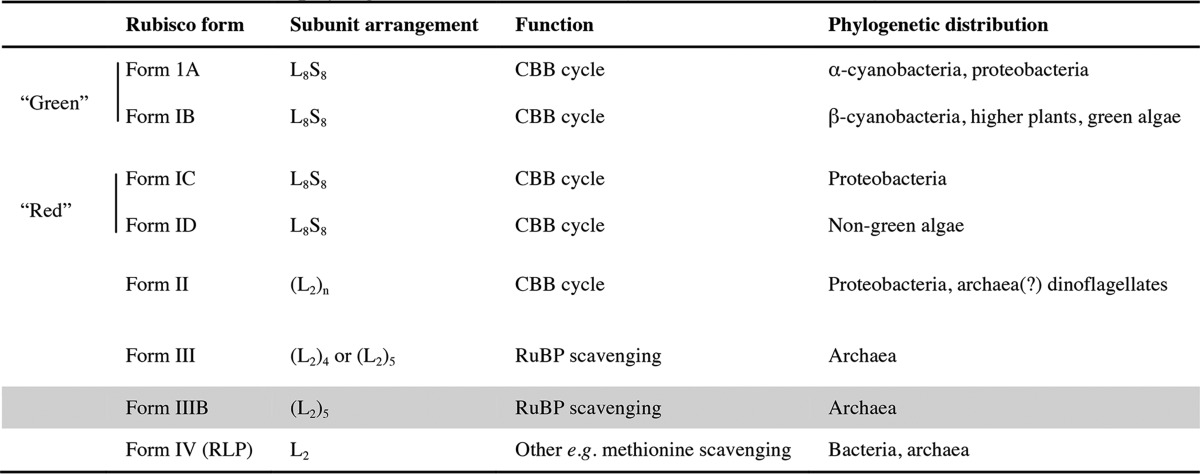
**Rubisco classification** Rubisco isoforms are currently divided into four classes (form I-III, RLP). The form I division is further divided into four subclasses based on sequence homology, subunit arrangement, and function. The newly proposed Rubisco subgroup, form IIIB, is shaded grey. All form IIIB Rubiscos are found in archaeal species, contain the Rubisco assembly domain that facilitates LSu oligomerization, function to scavenge RuBP, and exhibit distinct phylogenetic characteristics (see Fig. 6 and supplemental Fig. S3).

The overall MbR LSu structure is highly similar to form II and form III Rubisco structures. The root mean square deviations (r.m.s.d.) between the Cα atoms of the MbR LSu and the LSus from the form II/III structures indicate that the MbR LSu is structurally more similar to the form II (mean r.m.s.d. 1.3) than the form III Rubisco LSus (mean r.m.s.d. 1.5), although the differences are small (Table 1). However, given the limited availability of structural data for form II and form III Rubiscos, the relatively low resolution of the majority of the available structures, and the small structural differences, overall, the LSu structural divergence as evaluated by r.m.s.d. is insufficient to convincingly classify MbR as a form II or form III Rubisco.

The most prominent difference between MbR and other Rubisco structures is the presence of the assembly domain, which is unique to MbR. Apart from an additional N-terminal helix, unique to MbR, the MbR N and C termini are otherwise structurally very similar to the form III structures (supplemental Fig. S2). Residues between αB and βA in the N-terminal domain, which fold as a helix in the proteobacterial structures, form an unstructured loop in the MbR and archaeal structures (region *i* in supplemental Figs. S1 and S2), and the form II Rubisco structures are extended by two short extra helices at the C terminus that are not present in the form III or MbR structures (region *viii* supplemental Figs. S1 and S2).

Subtle, yet consistent, differences between the form II and form III Rubisco structures may be significant: variation in the lengths and conformations of the loops that connect secondary structure elements are evident in both sequence and structural alignments (regions *i–viii* in supplemental Figs. S1 and S2). The MbR LSu exhibits distinctly form II-like structure in βB-βC, α3-β4, αI-βE, and βF-βG (supplemental Fig. S2*F*). Loop regions in which the MbR LSu assumes a form III-like structure include the loop before β1 in the C-terminal domain and ηA-αE (supplemental Fig. S2*F*). Loop 6 assumes different conformations in the different structures, presumably as a consequence of the character of the ligand bound in the active site in the various structures. We conclude that the MbR LSu structure exhibits the loop length and positioning characteristic of either the form II or form III Rubiscos in different loops. The archaeal ancestry of MbR is distinct, and MbR appears to occupy an intermediate sequence and structural position between the proteobacterial form II and archaeal form III Rubiscos.

In general, *R. rubrum* Rubisco is structurally more similar to the form II than form III Rubiscos, as illustrated by the structural superposition (Table 1 and supplemental Fig. S2). Whereas the N-terminal domain and the bulk of the βα-barrel in the LSu of the neighboring barrel superimpose almost perfectly, the C-terminal region of *R. rubrum* Rubisco is shifted with respect to both form II and form III structures; this shift includes helix 7 and helix 8 of the βα-barrel and the remaining C-terminal region. In this respect, the *R. rubrum* structure appears to be a structural outlier within the form II/III groups. A similar observation was also made in a comparison with the form I spinach enzyme (28).

### Distribution of the assembly domain sequence

The length of the RAD sequence (29 amino acids) complicates the identification of sequences that exhibit statistically convincing homology. A BLAST search of the NCBI database yields no protein sequences with Expect values (E) below 0.1 that exhibit similarity to the assembly motif sequence from MbR, apart from a subset of Rubiscos from the Methanosarcinales order (sequences marked with an *asterisk* in supplemental Table S2 and a *gray arrow* in supplemental Fig. S3). The MbR RAD exhibited the highest sequence identity (90%) to the RAD from *Methanococcoides methylutens*. Although there were many hits to the full MbR sequence in metagenomic databases (data not shown), no significant hits were obtained for the assembly motif sequence alone (all hits with E values <10 are listed in supplemental Table S1), apart from one hit (E = 0.046) to the assembly motif from *Methanosaeta concilii* (see supplemental Table S2). The evolutionary acquisition of the assembly domain sequence thus remains unclear.

**Figure 6.**
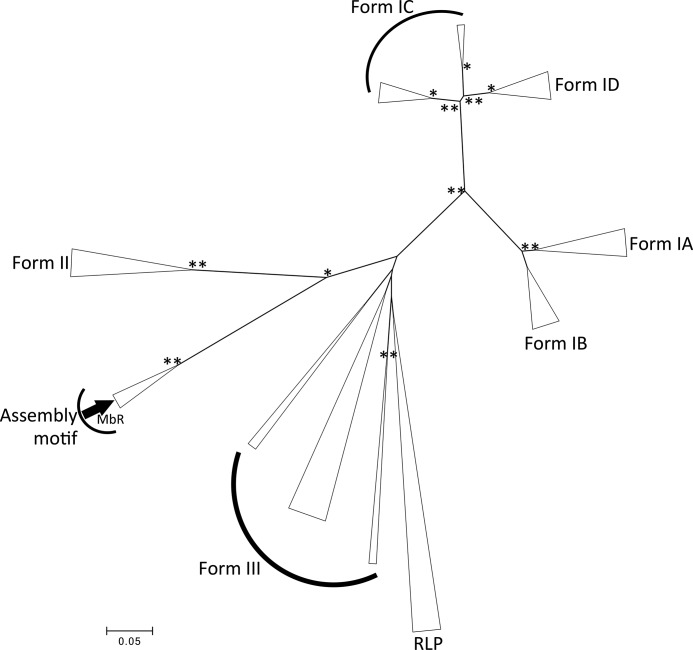
**Unrooted minimum evolution phylogenetic tree of Rubisco LSu sequences.** The optimal unrooted Rubisco LSu tree with the sum of branch length = 12.58 is shown. The tree is drawn to scale, with branch lengths in the same units as those of the evolutionary distances used to infer the phylogenetic tree. The evolutionary distances are in the units of the number of amino acid differences per site. The analysis included 15 representative sequences from each of the forms IA–ID, form II, form III, and form IV (RLPs) Rubisco groups and subgroups and all available Rubisco sequences from the Methanosarcinales order. The sequences used for phylogenetic reconstruction, and their homology to MbR, are included in supplemental Table S2. Bootstrap values ≥95% (** = 100%, * = 95–99%) obtained after 2000 bootstrap iterations are plotted at branch points. A *black arrow* indicates the location of the MbR sequence.

The phylogenetic tree showing the evolutionary history of the Rubisco LSu in Fig. 6 is congruous with the Rubisco distribution reported in previous studies (7, 17, 29). Form I Rubiscos and their subgroups cluster as a lineage distinct from other Rubisco forms. Although there are bootstrap values less than 95% for branch points leading to the form II, form III, and Rubisco-like protein (RLP) divisions, individual clades within these divisions are statistically convincing (supplemental Fig. S3). Despite overall uncertainty about the evolutionary history of the form III Rubiscos, the form II and RAD Rubisco lineages clearly diverge from the form III sequences.

All Rubisco sequences within the Methanosarcinales order that contains an assembly domain sequence (labeled *Assembly motif* in Fig. 6) share a common ancestor with the form II Rubiscos, accounting for the RAD Rubiscos exhibiting the highest sequence similarity to form II Rubiscos. However, the RAD Rubisco clade is distinct from the form II Rubiscos. Because all positions in the multiple sequence alignment that contained gaps were deleted prior to phylogenetic analyses, the assembly domain sequence does not contribute to the tree construction, and thus the phylogenetic relationship between the RAD Rubiscos runs much deeper than simply the presence of the additional sequence.

Most Rubisco sequences lacking the assembly domain sequence from the order Methanosarcinales cluster together in a clade within the form III Rubiscos (Fig. 6, *gray arrows*, also see supplemental Fig. S3) with 100% bootstrap confidence. The exceptions are the Rubisco sequences from *Methanoseta harundinacea* and *Methanoseta thermophila*, which cluster together nearby with Thermoplasmatales and Methanomassiliicoccales form III Rubiscos. The Candidatus *Methanoperedens nitroreducens* Rubisco sequence clusters with Methanomicrobiales form III Rubiscos. It is not surprising that closely related organisms may have divergent Rubisco sequences, especially given the rampant horizontal gene transfer of Rubisco-encoding genes. Rubisco phylogeny is often incongruent with phylogenies created using non-Rubisco-encoding genes (29, 31).

*Methanomethylovorans hollandica* appears to harbor two distinct Rubisco-encoding genes annotated in this study as “*M. hollandica (A*)” (MhR-A) and “*M. hollandica (B*)” (MhR-B) (supplemental Table S2). MhR-B contains an assembly domain sequence, whereas MhR-A does not. The full MhR-A and MhR-B sequences exhibit only 32% identity. When the RAD sequences are excluded from analyses, MhR-A and MhR-B exhibit 31% sequence identity. Thus, just like Methanosarcinales Rubiscos with and without the RAD sequence (supplemental Fig. S3), the MhR-A and MhR-B sequences differ much more fundamentally than just the presence/absence of the assembly domain sequence. MhR-A branches early from all the Methanosarcinales Rubiscos lacking the RAD sequence with 100% bootstrap confidence. The function of both Rubisco isoforms present in *M. hollandica* have not been studied, and thus it is not known whether MhR-A and MhR-B have distinct functional roles or how *M. hollandica* acquired two distinct Rubisco isoforms. Further work is required to find the evolutionary origin of the RAD Rubiscos. It is conceivable that a duplication event and subsequent divergence of one of the copies of Rubisco in an archaeal species could have given rise to the RAD Rubiscos.

### Rubisco sequence identity and Rubisco classification

MbR exhibits up to 41% identity to form II Rubiscos and up to 37% to the form III Rubiscos (supplemental Table S2). However, using this small difference in sequence similarity is not a very convincing basis for Rubisco classification as a form II Rubisco. In fact, Methanosarcinales RAD Rubiscos share similar amino acid identities with the form II proteobacterial (36–41% identity) and the form III archaeal Rubiscos (33–37% identity), but those without a RAD sequence are more similar to the form III (35–52% identity) than form II Rubiscos (25–33% identity; supplemental Table S2). The MbR sequence was compared with all archaeal Rubisco sequences available in the NCBI database (831 sequences). MbR is much more similar to RAD Rubiscos, exhibiting ≥74% sequence identity with archaeal Rubiscos that do contain an assembly domain sequence and ≤38% identity with those that do not contain an assembly domain sequence (data not shown).

## Discussion

### Re-evaluating Rubisco classification

It is not possible to clearly classify MbR as a form II or form III Rubisco. MbR's initial classification as a form II enzyme based on sequence homology (7, 32) was appropriate given the available information, but the validity of this classification has been questioned by other researchers (10) and warrants reconsideration especially now that new structural information has been gathered.

Under the current classification system, form I and II Rubiscos are photosynthetic; RLPs are strictly non-photosynthetic; and form III Rubiscos are found in non-photosynthetic organisms but can functionally substitute for form I and II Rubiscos *in vivo* (Table 2). MbR scavenges the RuBP by-product of purine/pyrimidine metabolism in *M. burtonii* but can also catalyze the addition of CO_2_ to RuBP when transplanted into photosynthetic organisms. Thus, despite the MbR amino acid sequence more closely resembling certain form II Rubiscos, the function of MbR is characteristic of form III Rubiscos. MbR kinetics are intermediate between the form II and form III Rubiscos. Different kinetic properties of MbR are either more similar to the form II (*e.g.* affinity for CO_2_) or form III Rubiscos (*e.g.* a much higher affinity for substrate RuBP consistent with its function recycling RuBP present in low abundance (10)). Uniquely, MbR exhibits high affinity for O_2_ and a much lower rate of oxygenation, which may be explained by the anoxygenic environment of *M. burtonii*.

MbR's subunit arrangement as an oligomer of dimers is common to form II and form III Rubiscos (Table 2). MbR has a unique assembly domain, which corresponds to a unique structural variation (Fig. 1). Unique structural features can be used to clearly distinguish between Rubisco forms, *e.g.* a βE-βF loop is only present in the SSu of “red” Rubiscos (form IC and ID, see Table 2). Similarly, common structural variation can be used to distinguish between distinct Rubisco subgroups, *i.e.* the length of the βA-βB loop that varies in the Rubisco SSu of form I Rubiscos (33–35).

Sequence comparisons of Methanosarcinales Rubiscos with and without the RAD suggest that the forms lacking the RAD are clearly form III, but the Rubiscos containing the RAD cannot be clearly determined to be form II or form III Rubiscos based on sequence homology alone. Furthermore, MbR is phylogenetically distinct from both the archaeal and proteobacterial Rubiscos (Figs. 6 and supplemental Fig. S3).

We propose that, based on MbR sequence, subunit arrangement and structure, and phylogenetic distribution and function, MbR and a subset of Rubiscos from the Methanosarcinales order belong to a new Rubisco subgroup, form IIIB. We favor introducing a new subclass of form III Rubiscos (rather than form II) on the basis of function.

### Rubisco and inhibitory ligands

The inhibition of form I Rubisco activity upon binding of XuBP and other 6-carbon molecules is well studied (36). XuBP is a more potent inhibitor of higher plant Rubiscos than other form I Rubiscos (37). XuBP can also weakly bind *R. rubrum* Rubisco (38) and non-photosynthetic RLPs (39). No data for XuBP binding to any form III Rubiscos are available. Photosynthetic Rubiscos, in particular those from higher plants, may in fact be adapted to bind inhibitors, *i.e.* the Rubisco active site binding inhibitory sugars is not promiscuity on behalf of Rubisco, but rather is a selected adaptation. Tight binding of inhibitory sugars to higher plant Rubiscos necessitates a heavy reliance on complicated regulatory pathways to remove these compounds (36). Rubisco inhibitors are produced under conditions where it is less than optimal for high rates of Rubisco catalysis, for example in the dark in the absence of energy production to drive the Calvin-Benson-Bassham cycle. In organisms with a form I Rubisco, an ATP-driven enzyme, Rubisco activase, actively removes inhibitory sugars (40, 41). Thus, binding of inhibitory sugars results in tighter control of Rubisco activity to ensure that Rubisco is only active when it is required.

### RAD as a Rubisco concentrating mechanism

MbR has its own inbuilt SSu mimic that concentrates L_2_ dimers by the coordination of residues with negatively charged side chains around Mg^2+^. This unique structural variation has not been observed in any Rubisco crystal structure to date. Mechanisms that concentrate Rubisco are widespread in nature, suggesting that they confer some advantage, *e.g.* SSus are required for optimal activity of form I Rubiscos, higher order form III Rubisco complexes exhibit increased thermostability, and the essential pyrenoid component 1 protein concentrates and localizes Rubisco to *Chlamydomonas* pyrenoids (42). Rubisco SSus sequester and concentrate LSu dimers, and carbon concentrating mechanisms, such as carboxysomes in cyanobacteria and altered leaf physiology in C_4_ plants, not only concentrate but also act to compartmentalize Rubisco such that the relative concentrations of substrate CO_2_ and inhibitory O_2_ can be controlled. Although the Rubisco SSu shows sequence homology to one of the carboxysome shell proteins, the origin of the MbR assembly motif sequence is not clear; this sequence exhibits statistically weak homology to proteins of bacterial, viral, and archaeal origin. The RAD may have evolved completely independently of other Rubisco-concentrating mechanisms.

It is feasible that substrate binding at the Rubisco active site could induce structural rearrangements bringing the αJ helix into closer proximity to helix α1 for the lock site to form. Although the structural data presented here provides clues as to how these messages could be communicated, there is yet insufficient data to propose a mechanism. The structure of the assembly domain in dimeric L_2_ MbR is unknown. The RAD could be flexible and undergo a large structural change upon substrate binding or the conformational differences between the L_2_ and L_10_ form could be more subtle.

The structural characterization of the assembly domain from MbR offers exciting opportunities to use this structural element in engineering strategies; the domain could be used as a protein glue to force protein units to associate, particularly in a Mg^2+^ (or other ion)-dependent manner. Recently, there has been a lot of interest in introducing carboxysomes into higher plant chloroplasts to concentrate Rubisco and occlude inhibitory O_2_ to improve the catalytic performance of Rubisco (43). The assembly domain could provide another route to concentrate Rubisco LSus, particularly in variants engineered from the more primitive form II or form III Rubisco templates via rational design or directed evolution approaches.

## Experimental procedures

### DNA cloning

The pHUE-MbiiL plasmid (10) that encodes the *M. burtonii* Rubisco LSu immediately downstream of His_6_-ubiquitin (H_6_-Ub) in the pHue vector (44) was used as the template to introduce single site-specific mutations into MbR. The following primers were designed to introduce these mutations using the QuikChange II (Agilent) mutagenesis kit: E179A (5′-CCTCAGCGGAATATGCGGCAGTATGTTATGATTTCTG-3′); E183A (5′-GGAATATGCGGAAGTATGTTATGCTTTCTGGGTAGGTGG-3′); D366A (5′-GTCAAAGATAATGGATACCGCCAAGGATGTCATCAACCTTG-3′); K367A (5′-TGGTCAAAGATAATGGATACCGACGCCGATGTCATCAACCTTGTTAATGAG-3′); and D368A (5′-GATAATGGATACCGACAAGGCTGTCATCAACCTTGTTAATG-3′). Similarly, to introduce double mutations into MbR, the D183A primer was used to introduce a D183A mutation into the pHUE-MbiiL-D366A and pHUE-MbiiL-D368A plasmids created as described above. The primer D366A_D368A (5′-GATAATGGATACCGACAAGGCTGTCATCAACCTTGTTAATG-3′) was used to introduce a D368A mutation into the pHUE-MbiiL-D366A plasmid. All changes are underlined. Incorporation of the desired mutations within the coding region of modified plasmids was verified by BigDye terminator sequencing with a forward primer near the 3′ end of Ub, a pET-reverse primer (45), and an additional internal sequencing primer, MbRaF (5′-GATGGGACTTACCTCAGCGG-3′). DNA sequencing was performed by SciLifeLab (Uppsala, Sweden).

### Protein expression

The pHUE-MbiiL plasmid and mutated derivative plasmids were used to express MbR in *Escherichia coli* BL21 cells (Thermo Fisher Scientific). *E. coli* cells were grown in 4 liters (pHUE-MbiiL for crystallization) or 50 ml (mutant constructs for oligomerization experiments) of 2× YT medium (1.6% tryptone, 1% yeast extract, 0.5% NaCl) with 200 μg ml^−1^ ampicillin at 37 °C. Cells were cultured to mid-log phase and chilled on ice, and then protein expression was induced by addition of 0.5 mm isopropyl β-d-1-thiogalactopyranoside and incubation for 4 h at 30 °C. H_6_-Usp2cc was expressed and purified as described previously (44).

### Protein purification

MbR for crystallographic study and ligand-binding experiments was purified using a multistep purification procedure that involved immobilized metal affinity chromatography (IMAC), H_6_-Ub tag cleavage, and removal and size-exclusion chromatography.

Cells were resuspended in cell lysis buffer (50 mm Tris-Cl, pH 8.0, 300 mm NaCl, 10 mm imidazole, 2 mm MgCl_2_, protease inhibitor tablets (Pierce), 1 μg ml^−1^ DNase I, 5% glycerol) and lysed by sonication. Soluble protein was obtained by centrifugation (48,000 × *g*, Beckman JL-25.50), applied to 5 ml of Profinity^TM^ IMAC resin (Bio-Rad), and allowed to enter the resin by gravity flow. The resin was washed extensively with Wash buffer (50 mm Tris-Cl, pH 8.0, 300 mm NaCl, 10 mm imidazole), and protein was eluted using Elution buffer (Lysis buffer supplemented with 240 mm imidazole). Peak fractions were pooled, and the H_6_-Ub tag was cleaved with H_6_-Usp2cc, as described previously (45), where precise H_6_-Ub tag cleavage yields an unmodified MbR protein product.

Protein was buffer exchanged overnight at 4 °C into Superdex buffer (100 mm Bicine, pH 8.0, 50 mm NaCl, 10 mm MgCl_2_, 1 mm EDTA), and then the H_6_-Ub tag, uncleaved protein, and H_6_-Ub-Usp2 were selectively removed by passage through Superdex buffer-equilibrated Profinity^TM^ IMAC.

A final purification step was performed at 4 °C by size-exclusion chromatography using a HiLoad 26/60 Superdex 200 (GE Healthcare) column with a flow rate of 2 ml min^−1^ attached to a NGC chromatography system (Bio-Rad). Peak fractions were evaluated by non-denaturing and SDS-PAGE, pooled, and dialyzed into Crystallization buffer (100 mm HEPES-OH, pH 8.0, 10 mm MgCl_2_, 1 mm EDTA, 10 mm NaHCO_3_).

Mutated MbR protein for oligomerization analyses was purified by IMAC as described above, except with only 1 ml of Profinity^TM^ IMAC resin (Bio-Rad). Purified Rubisco was activated by addition of 10 mm NaHCO_3_ (in addition to the 10 mm MgCl_2_ already present in the crystallization buffer) and incubated for 30 min at room temperature.

### Oligomerization experiments

The oligomerization capacity of MbR was determined in the presence of different ligands and in MbR harboring site-specific substitutions. Aliquots of activated purified soluble wild-type MbR were incubated with 1- (8 μm), 10- (80 μm), or 1000-fold (8 mm) molar concentrations (relative to the number of Rubisco active sites) of the ligands 3-PGA, XuBP, 4-CABP, or 2-CABP at room temperature for 1 h. Mutant MbR proteins were analyzed in the presence of a 10-fold molar excess of 2-CABP only. Addition of crystallization buffer was used as a negative control. 2-CABP was prepared as described previously (46). 4-CABP and XuBP were gifts from G. H. Lorimer (Department of Chemistry and Biochemistry, University of Maryland). To chelate the Mg^2+^ bound at the dimer-dimer lock site, 2-CABP-bound wild-type and mutant MbR were incubated with equimolar (20 mm) or 2× molar (40 mm) concentrations of EDTA (relative to Mg^2+^ concentration in the buffer) for 30 min at room temperature.

### PAGE analyses

Proteins were separated on 4–15% Mini-Protean TGX Stain-Free gels (Bio-Rad) in a Mini-Protean Tetra Vertical Electrophoresis Cell (Bio-Rad). Non-denaturing (native)-PAGE was run in native running buffer (25 mm Tris, 0.19 m glycine, pH 8.3). For SDS-PAGE separation, the native running buffer was supplemented with 1% SDS. PAGE-separated proteins were visualized by staining with AcquaStain (Bulldog Bio). Precision Plus Protein Unstained Protein Marker (Bio-Rad) or High Molecular Weight Native Marker (GE Healthcare) were included for size comparisons.

### Crystallization

A 10-fold molar excess of 2-CABP was added to purified and activated MbR and incubated for 1 h at room temperature to induce oligomerization (10). (L_2_)_5_-MbR was then concentrated to 5 mg ml^−1^ (Vivaspin, Sartorius Stedim Biotech) in Crystallization buffer (100 mm HEPES-OH, pH 8.0, 10 mm MgCl_2_, 1 mm EDTA, 10 mm NaHCO_3_). Equal volumes of (L_2_)_5_-MbR were mixed with the reservoir solution (0.1 m Tris, pH 7.8, 0.2 m KBr, 2.9% polyglutamic acid (w/v), 4.8% PEG 20K (w/v)). A purified and concentrated aliquot of MbR was also incubated with a 10-fold molar excess of XuBP and diluted to 4 mg ml^−1^ in Crystallization buffer before equal volumes of L_2_-MbR were mixed with the reservoir solution (0.1 m Tris, pH 7.0, 0.2 m l-arginine, 15% PEG 3350 (w/v)). Crystals were obtained by the hanging-drop vapor-diffusion method after equilibration at room temperature for 1–3 weeks. In preparation for data collection, crystals were transferred to a nylon loop (Hampton Research), soaked in cryo-protectant solution (reservoir solution containing 25% ethylene glycol), and then flash-cooled in liquid nitrogen.

### 2-CABP-bound MbR data collection and structure determination

X-ray diffraction data were collected on beamline ID-29 of the European Synchrotron Radiation Facility (ESRF), Grenoble, France, at 100 K with a Pilatus 6M pixel detector. Data were processed using XDS (47) and scaled and merged using AIMLESS (48, 49), with 5% of reflections set aside for calculation of free *R* values. Calculation of the Matthews coefficient (*V_M_* = 3.96 Å^3^ Da^−1^ (50)), assuming an LSu molecular mass of 52,857 Da (10), predicted the presence of half a decamer (L_2_)_5_, per asymmetric unit, corresponding to a solvent content of 69%. The Diffraction Anisotropy Server at UCLA (51) indicated that there was almost no anisotropy. Recommended resolution limits where *F*σ was above 3.0 along *a*, *b*, and *c* were 2.6, 2.6, and 2.7 Å, respectively. Despite high *R*_merge_ and low *CC*_1/2_, *I*/σ remained above 1.0 down to a resolution of 2.6 Å. Reflections were therefore used from 48.3 to 2.6 Å. Phases were obtained by molecular replacement using Phaser (52, 53) with one L_2_ dimer from the X-ray crystal structure of *R. palustris* Rubisco (PDB code 4LF1) as the search model. Structures were obtained by alternating between refinement using BUSTER (54) and manual building in Coot (55) and O (56). Waters were inserted by alternating between automated water addition using Phenix refine (53, 57) and manual evaluation in Coot. The final model has an *R*_work_ and *R*_free_ of 0.1890 and 0.2250, respectively, for all data between 48.37 and 2.6 Å. Data collection and refinement statistics are summarized in Table 3. Ramachandran plots were calculated using the PDB Server (58) to assess model geometry and indicated that 1.0% of the residues lie in disallowed regions. These residues were located in well defined density and exhibit no direct or obvious involvement in MbR catalysis or assembly. The mean coordinate error was 0.340 Å as calculated from a Luzzati plot.

**Table 3:** **Data collection and refinement statistics for MbR** Values in parentheses are for the outer shell.

**Data collection**	
Beamline	ID29, ESRF
Wavelength (Å)	0.9763
Space group	*P*321
Unit cell parameters (Å, °)	*a* = *b* = 273.8, *c* = 96.7, γ = 120.0
Resolution range (Å)	48.4–2.6 (2.69–2.60)
Total no. of observations	1,293,072 (49,370)
No. of unique reflections	126,745
*R*_meas_*^a^*	0.238 (2.059)
*I*/σ(*I*)	10.8 (1.3)
*CC*_1/2_*^b^*	0.994 (0.231)
Completeness (%)	99.7 (93.7)
Multiplicity	10.2 (8.5)

**Refinement**	
Resolution range (Å)	48.4–2.6 (2.65–2.60)
No. of reflections	12,6739
*R*_cryst_*^c^*	0.189
R_free_*^c^*	0.225
Residues in model	A1–473, B1–473, C1–472, D1–473, E2–473
No. of atoms	19,003
Protein	18,589
Waters	294
Mg^2+^	10
Cl^−^	5
2-CABP	105
Average *B*-values (Å^2^)	
Estimated from Wilson plot	78.8
r.m.s. deviations from ideal values	
Bond lengths (Å)	0.010
Bond angles (°)	1.29
Ramachandran analysis*^d^*	
Outliers (%)	1.0

*^a^* Data are as defined by Diederichs and Karplus (69).

*^b^ CC*_1/2_ was calculated by first randomly splitting the unmerged data into two, and then calculating the Pearson correlation coefficient between the average intensities of these two data sets (30).

*^c^ R*_cryst_ = Σ*_hkl_*‖*F*_obs_| − ‖*F*_calc_‖/Σ*_hkl_* |*F*_obs_|, where *F*_obs_ and *F*_calc_ are the observed and calculated structure factor amplitudes, respectively. *R*_free_ was calculated from 5% of randomly selected unique reflections.

*^d^* From PDB Server (58).

### XuBP-bound MbR data collection and structure determination

X-ray diffraction data were collected on beam line ID30A-3 of the ESRF at 100 K with a Pilatus3X 2M pixel detector. Data processing, scaling, and merging was performed as described for the 2-CABP-bound structure above. The 2-CABP-bound MbR crystal structure was used as the search model to obtain the XuBP-incubated MbR phases.

### Sequence and structure comparison

The full MbR amino acid sequence (containing the assembly motif, residues 361–389) was compared with other sequences using the “Protein Sequences for Metagenomes” database from NCBI. Also searched were the ALV, AMB, BHA, BKD, DMB, EKJ, FRI, GAI, GCH, GCM, HFG, NXV, TXW, UGW, and WFB databases from MetagenomesOnline (59). Databases were selected to cover a range of different environments, organisms, and geographical locations. Sequence alignments were generated using BlastP 2.3.0+ (60) employing composition-based statistics (61) using the default search strategy (*e.g.* BLOSUM62 matrix) for full Rubisco sequences and the preset search strategy optimized for short sequences (*e.g.* PAM30 matrix) for assembly motif alignments.

A structural superposition of MbR with bacterial Rubisco structures in the PDB was performed with the least squares superposition function in O (56). The default distance cutoff limit of 3.8 Å was used. Amino acid sequence alignments were created using ClustalOmega (62) and manually adjusted to reflect the structural alignment obtained in O. The graphical output was created in ESPript (63). The MbR structure was also compared with other structures available in the PDB using the Dali server (18). The assembly domain was defined as residues 360–390 for Dali structural analysis because this resource requires a query structure with a minimum of 30 amino acids.

### Phylogenetic analyses

A Rubisco LSu multiple sequence alignment was generated in COBALT (64), and evolutionary analyses were conducted in MEGA6 (65). The analysis involved 128 amino acid sequences. All positions containing gaps and missing data were eliminated. There were a total of 282 positions in the final dataset. The evolutionary history of the Rubisco LSu was inferred using the Minimum Evolution method (66). The evolutionary distances were computed using the p-distance method (67). The Minimum Evolution tree was searched using the Close-Neighbor-Interchange algorithm (67) at a search level of 1. The Neighbor-joining algorithm (68) was used to generate the initial tree. Bootstrap values were obtained from 2000 bootstrap iterations.

### Electrostatic surface analysis

Electrostatic potentials were calculated in the PyMOL Molecular Graphics System (Version 1.7.4, Schrödinger, LLC) using the generate vacuum electrostatics function. Analyses were performed on the coordinates of one Rubisco L_2_ dimer from *T. kodakarensis* (PDB code 1GEH) and MbR. All solvent molecules were removed before analysis.

### Other software

Figures were produced using PyMOL.

## Author contributions

L. H. G. and I. A. conceived the study, wrote the paper, and analyzed the data. L. H. G., I. A., and K. V. undertook crystal structure refinement and interpretation of the electron density. I. A. performed sequence-structural alignments. L. H. G. designed and conducted the experiments. All authors reviewed the results and approved the final version of the manuscript.

## Supplementary Material

Supplemental Data

## Footnotes

2 The abbreviations used are:

RuBP: ribulose 1,5-bisphosphate
Rubisco: RuBP carboxylase/oxygenase
3-PGA: 3-phosphoglycerate
LSu: Rubisco large subunit
SSu: Rubisco small subunit
MbR: *M. burtonii* Rubisco
RAD: Rubisco assembly domain
2-CABP: 2-carboxyarabinitol 1,5-bisphosphate
4-CABP: 4-carboxyarabinitol 1,5-bisphosphate
XuBP: xylulose 1,5-bisphosphate
PDB: Protein Data Bank
r.m.s.d.: root mean square deviation
E: expect value
RLP: Rubisco-like protein
MhR: *M. hollandica* Rubisco
H_6_-Ub: His_6_-ubiquitin
ESRF: European Synchrotron Research Facility
Bicine: *N*,*N*-bis(2-hydroxyethyl)glycine
IMAC: immobilized metal affinity chromatography.
