# Supplementary material for: A unique structural domain in Methanococcoides burtonii ribulose-1,5-bisphosphate carboxylase/oxygenase (Rubisco) acts as a small subunit mimic
Source: J Biol Chem. 2017 Jan 30;292(16):6838–50. doi: 10.1074/jbc.M116.767145 (PMC5399129; doi:10.1074/jbc.M116.767145)
Supplement: Supplemental Data [file supp_292_16_6838__index.html]

A Unique Structural Domain in Methanococcoides burtonii Rubisco Acts as a Small-subunit Mimic — A unique structural domain in Methanococcoides burtonii ribulose-1,5-bisphosphate carboxylase/oxygenase (Rubisco) acts as a small subunit mimic — M. burtonii Rubisco structure and oligomerization — Supplemental Data 

# A unique structural domain in *Methanococcoides burtonii* ribulose-1,5-bisphosphate carboxylase/oxygenase (Rubisco) acts as a small subunit mimic

## Supplemental Data

- All supplemental data (.pdf, 6.2 MB) - All supplemental data: supplemental Table S1 (metagenomics), Table S2 (sequences), Fig. S1 (sequence and structure alignment), Fig. S2 (structure alignment) and Fig. S3 (phylogenetics).
