# Supplementary material for: A unique structural domain in Methanococcoides burtonii ribulose-1,5-bisphosphate carboxylase/oxygenase (Rubisco) acts as a small subunit mimic
Source: J Biol Chem. 2017 Jan 30;292(16):6838–50. doi: 10.1074/jbc.M116.767145 (PMC5399129; doi:10.1074/jbc.M116.767145)
Supplement: Supplemental Data [file 10.1074_M116.767145_jbc.M116.767145-1.pdf]

SUPPLEMENTAL DATA

A unique structural domain in *Methanococcoides burtonii* Rubisco acts as a small-subunit mimic.

Laura H. Gunn, Karin Valegård, Inger Andersson

SUPPLEMENTAL TABLE S1. Summary of sequences from metagenomic databases that exhibit homology to the Rubisco assembly motif. All significant alignments between the MbR assembly motif and sequences from a diverse range of metagenomic databases are shown. One hit with an E Value < 0.1 is shaded grey, and corresponds to the assembly motif in the Rubisco isoform from *Methanosaeta concilii*.

Identifier	Project	Location	Type	Sphere	Ecosystem	Sequences producing significant alignments:	Score (Bits)	E Value	Identities	Positives	Gaps
ALV	Alvinella pompejana Epibiont Metagenome	East Pacific Rise	Bacterial/Archeal dsDNA	Organismal	Vent/Seep	None					
AMB	Acid Mine Drainage Metagenome	Richmond Mine	Bacterial/Archeal dsDNA	Aquatic	Subterranean	AMB_JCVI_PEP_1113367531784 ACID_MINE_05	25.7	5.2	11/17 (65%)	11/17 (65%)	1/17 (6%)
						AMB_JCVI_PEP_1113951729816 ACID_MINE_05	25.2	9.3	11/17 (65%)	1/17 (65%)	1/17 (6%)
BHA	GBMF-MVSP-J_Deming	Beaufort Sea	Viral DNA	Aquatic	Open Ocean	BHA_deg7180000007542_1_661_1	23.1	3.1	8/9 (89%)	8/9 (89%)	1/9 (11%)
BKD	GBMF-MVSP-J_Deming	Barrow Permafrost Shaft	Viral DNA	Aquatic	Tundra	BKD_deg7180000004248_1_220_1	21.0	5.7	10/21 (48%)	14/21 (67%)	2/21 (10%)
						BKD_ctg7180000004249_1_203_1	21.0	6.6	10/21 (48%)	14/21 (67%)	2/21 (10%)
						BKD_sngl100000120213_1_456_1	20.6	8.6	9/27 (33%)	9/27 (33%)	16/27 (59%)
DMB	Mediterranean Bathypelagic Habitat Metagenome	Ionian Km3 station	Bacterial/Archeal, Eukaryotic dsDNA	Aquatic	Open Ocean	DMB_JCVI_PEP_1113385913768 DEEPMED	22.7	3.0	7/10 (70%)	7/10 (70%)	2/10 (20%)
						DMB_JCVI_PEP_1113385927484 DEEPMED	21.4	8.3	6/8 (75%)	7/8 (88%)	0/8 (0%)
						DMB_JCVI_PEP_1113385908224 DEEPMED	21.4	8.9	6/8 (75%)	7/8 (88%)	0/8 (0%)
env_nr	NCBI- Protein sequences for metagenomes	various	various	various	various	gb|KUG17829.1| ribulose bisphosphate carboxylase	35.0	0.046	17/29 (59%)	19/29 (66%)	0/29 (0%)
						gb|EBP47145.1| hypothetical protein GOS_7905945	33.3	0.42	10/14 (71%)	11/14 (79%)	0/14 (0%)
						gb|EJW94105.1| 4-alpha-glucanotransferase	29.5	3.7	9/10 (90%)	9/10 (90%)	1/10 (10%)
						gb|EDJ37338.1| hypothetical protein GOS_1706882	28.6	6.1	9/15 (60%)	11/15 (73%)	0/15 (0%)
EKJ	GBMF-MVSP-DL_Valentine	Boiling Springs Lake	Viral DNA	Aquatic	Spring	None					
FRI	GBMF-MVSP-R_Danovaro	Arctic Ocean	Viral DNA	Aquatic	Open Ocean	FRI_sngl100000084480_188_455_2	22.7	5.7	9/16 (56%)	10/16 (63%)	6/16 (38%)
GAI	Global Ocean Sampling Expedition	Browns Bank, Gulf of Maine	Bacterial/Archeal dsDNA	Aquatic	Coastal	None					
GCH	Global Ocean Sampling Expedition	Indian Ocean	Bacterial/Archeal dsDNA	Aquatic	Open Ocean	GCH_JCVI_PEP_1112700407564 GS109	27.8	0.91	8/17 (47%)	13/17 (76%)	2/17 (12%)
						GCH_JCVI_PEP_1112700347868 GS109	26.9	1.9	7/8 (88%)	8/8 (100%)	0/8 (0%)
						GCH_JCVI_PEP_1112700540058 GS109	25.7	4.2	10/21 (48%)	12/21 (57%)	7/21 (33%)
						GCH_JCVI_PEP_1112700714730 GS109	24.8	7.2	7/10 (70%)	8/10 (80%)	0/10 (0%)
						GCH_JCVI_PEP_1112700405910 GS109	24.8	7.5	9/14 (64%)	10/14 (71%)	0/14 (0%)
						GCH_JCVI_PEP_1112700571250 GS109	24.8	7.9	7/10 (70%)	8/10 (80%)	0/10 (0%)
						GCH_JCVI_PEP_1112700369998 GS109	24.8	8.0	7/8 (88%)	7/8 (88%)	0/8 (0%)
						GCH_JCVI_PEP_1112700253142 GS109	24.4	9.6	7/10 (70%)	8/10 (80%)	0/10 (0%)
GCM	Global Ocean Sampling Expedition	Indian Ocean	Eukaryotic dsDNA	Aquatic	Open Ocean	GCM_JCVI_PEP_1112740922342 GS112b	25.7	5.1	9/16 (56%)	9/16 (56%)	0/16 (0%)
HFG	Microbial Community Genomics at the HOT/ALOHA	Hawaii Ocean Time-series station ALOHA	Bacterial/Archeal dsDNA	Aquatic	Open Ocean	HFG_JCVI_PEP_1114003973650 HF770_12_21_03	23.5	7.6	11/24 (46%)	14/24 (58%)	3/24 (13%)
NXV	GBMF-MVSP-RA_Sandaa	Raunefjorden	Viral DNA	Aquatic	Coastal	NXV_sngl100000073362_171_491_2	22.3	6.0	7/14 (50%)	10/14 (71%)	0/14 (0%)
TXW	GBMF-MVSP-M_Young	Yellowstone National Park	Viral DNA	Aquatic	Spring	None					
UGW	GBMF-MVSP-RL_Vega_Thurber	Pacific Ocean: Coral Sea	Viral DNA	Organismal	Coral Reef	None					
WFB	Whale Fall Metagenome	W. Antarctic Peninsula Shelf	Bacterial/Archeal dsDNA	Organismal	Open Ocean	WFB_JCVI_PEP_1113976024036 WHALEFALLBONE	26.5	0.72	9/16 (56%)	10/16 (63%)	5/16 (31%)
						WFB_JCVI_PEP_1113975968026 WHALEFALLBONE	24.0	6.7	7/9 (78%)	7/9 (78%)	0/9 (0%)
						WFB_JCVI_PEP_1113975991138 WHALEFALLBONE	23.5	7.5	6/8 (75%)	8/8 (100%)	0/8 (0%)
						WFB_JCVI_PEP_1113975920950 WHALEFALLBONE	23.5	9.9	12/24 (50%)	12/24 (50%)	4/24 (17%)

SUPPLEMENTAL TABLE *2*. Sequences used for phylogenetic analyses.
The accession numbers and source organism are shown for all 128 Rubisco sequences used for phylogenetic analyses (Figs. 6 and S3). The sequences are annotated with Rubisco group/subgroup and the percentage amino acid identity that each exhibits to MbR is also indicated. Rubiscos from the order *Methanosarcinales* are shaded grey. Rubisco sequences harboring the assembly motif are marked with an asterisk.

Accession number	Organism	Rubisco form	% sequence identity to MbR
AAK00291.1	*Acidithiobacillus ferrooxidans*	IA	31
AAA23328.1	*Allochromatium vinosum*	IA	32
EDY38204.1	*Cyanobium spp.* PCC 7001	IA	32
WP_011813845.1	*Halorhodospira halophila*	IA	31
AAC32549.1	*Halothiobacillus neapolitanus*	IA	30
BAD15312.1	*Hydrogenovibrio marinus*	IA	32
WP_010961949.1	*Methylococcus capsulatus*	IA	31
AAA25509.1	*Nitrobacter vulgaris*	IA	30
WP_011633896.1	*Nitrosomonas eutropha*	IA	30
ABS00401.1	*Paulinella chromatophora*	IA	31
AAC37141.1	*Rhodobacter capsulatus*	IA	31
ABI45662.1	*Synechococcus sp.* PCC 9311	IA	30
WP_011932588.1	*Synechococcus sp.* WH 7803	IA	30
AAB70697.1	*Thiobacillus denitrificans*	IA	31
WP_011369848.1	*Thiomicrospira crunogena*	IA	29
ABA23512.1	*Anabaena variabilis* ATCC 29413	IB	32
NP_958405.1	*Chlamydomonas reinhardtii*	IB	30
NP_045897.1	*Chlorella vulgaris*	IB	30
CAA37214.1	*Cyanophora paradoxa*	IB	31
NP_041936.1	*Euglena gracilis*	IB	30
NP_054507.1	*Nicotiana tabacum*	IB	30
NP_039391.1	*Oryza sativa*	IB	30
AGW31208.1	*Ostreococcus tauri*	IB	30
YP_008563096.1	*Solanum lycopersicum*	IB	30
NP_054944.1	*Spinacia oleracea*	IB	30
ABB57456.1	*Synechococcus elongatus* PCC 7942	IB	33
BAA10190.1	*Synechocystis sp.* PCC 6803	IB	32
BAC09058.1	*Thermosynechococcus elongatus* BP-1	IB	32
AHI44627.1	*Triticum aestivum*	IB	31
YP_636475.1	*Zygnema circumcarinatum*	IB	31
BAN85804.1	*Acidomonas methanolica*	IC	32
AAB41464.1	*Aurantimonas manganoxydans* SI85-9A1	IC	31
CUU41046.1	*Blastochloris viridis*	IC	32
GAJ33597.1	*Bradyrhizobium sp.* DOA9	IC	31
KGF69711.1	*Hoeflea sp.* BAL378	IC	33

WP_018632594.1	*Meganema perideroedes*	IC	32
CDX13026.1	*Mesorhizobium plurifarium*	IC	33
ABE64713.1	*Nitrobacter hamburgensis* X14	IC	32
WP_050474664.1	*Pannonibacter phragmitetus*	IC	32
WP_011747994.1	*Paracoccus denitrificans*	IC	32
WP_013651735.1	*Polymorphum gilvum*	IC	32
CAJ96184.1	*Ralstonia eutropha* H16	IC	33
AAA26115.1	*Rhodobacter sphaeroides*	IC	31
WP_037430179.1	*Sinorhizobium fredii*	IC	32
WP_012113941.1	*Xanthobacter autotrophicus*	IC	32
AKE98828.1	*Bangia fuscopurpurea*	ID	31
CCP38199.1	*Chondrus crispus*	ID	30
ADI87180.1	*Cumathamnion decipiens*	ID	30
CAA39140.1	*Cyanidium caldarium*	ID	30
AAF81681.1	*Galdieria sulphuraria*	ID	32
AFI24706.1	*Gracilaria chouae*	ID	31
AFI24726.1	*Gracilariopsis lemaneiformis*	ID	30
ABU53651.1	*Griffithsia monilis*	ID	30
AGH25102.1	*Kappaphycus alvarezii*	ID	31
ADU87388.1	*Laurencia intricata*	ID	31
AAO49751.1	*Mastocarpus papillatus*	ID	31
AET10441.1	*Phaeodactylum tricornutum*	ID	31
NP_053836.1	*Porphyra purpurea*	ID	31
ADI87209.1	*Ptilota filicina*	ID	30
YP_007947755.1	*Pyropia haitanensis*	ID	31
BAF61766.1	*Candidatus Vesicomyosocius okutanii* HA	II	38
WP_027191928.1	*Desulfovibrio putealis*	II	40
EGV51960.1	*endosymbiont of Riftia pachyptila*	II	40
AAD02442.1	*Halothiobacillus neapolitanus* c2	II	38
WP_011385076.1	*Magnetospirillum magneticum*	II	39
WP_002725205.1	*Phaeospirillum molischianum*	II	40
AAB82048.1	*Rhodobacter capsulatus*	II	41
WP_011339170.1	*Rhodobacter sphaeroides*	II	40
WP_011463692.1	*Rhodoferax ferrireducens*	II	38
4LF1_A	*Rhodopseudomonas palustris*	II	40
ABC23200.1	*Rhodospirillum rubrum* ATCC 11170	II	39
CUH64377.1	*Thalassobius gelatinovorus*	II	39
WP_011313150.1	*Thiobacillus denitrificans*	II	38
WP_028488884.1	*Thiothrix lacustris*	II	38
5C2C_A	*Uncultivated Gallionellacea species*	II	38
WP_008086508.1	*Aciduliprofundum boonei*	III	38
KUJ93020.1	*Archaeoglobus fulgidus*	III	33
WP_048113196.1	*Candidatus Methanoplasma termitum*	III	31

	WP_050048430.1	*Halanaeroarchaeum sulfurireducens*	III	35
	WP_049984881.1	*Halobellus rufus*	III	33
	WP_049911711.1	*Haloterrigena limicola*	III	34
	WP_011821683.1	*Hyperthermus butylicus*	III	38
	B64454	*Methanococcus jannaschii*	III	35
	WP_014868062.1	*Methanoculleus bourgensis*	III	36
	WP_015286150.1	*Methanoregula formicica*	III	34
	2D69_A	*Pyrococcus Horikoshii*	III	34
	WP_011752890.1	*Thermofilum pendens*	III	33
	WP_015491731.1	*Thermoplasmatales archaeon* BRNA1	III	33
	WP_013129771.1	*Thermosphaera aggregans*	III	34
	1GEH_A	*Thermosynechococcus kodakarensis*	III	33
	WP_048093891.1	*Candidatus Methanoperedens nitroreducens*	III	32
	ADI73811.1	*Methanohalobium evestigatum* Z-7303	III	37
	WP_015325018.1	*Methanomethylovorans hollandica* (A)	III	33
	WP_014587790.1	*Methanosaeta harundinacea*	III	33
	ABK15383.1	*Methanosaeta thermophila* PT	III	35
	AKB82927.1	*Methanosarcina barkeri 3*	III	37
	KPL45065.1	*Methanosarcina flavescens*	III	36
	AKB76558.1	*Methanosarcina horonobensis* HB-1 = JCM 15518	III	37
	AKB73426.1	*Methanosarcina lacustris* Z-7289	III	36
	AKB39058.1	*Methanosarcina mazei* WWM610	III	36
	WP_048174263.1	*Methanosarcina siciliae*	III	36
	KKG14490.1	*Methanosarcina sp.* 2.H.T.1A.3	III	36
	AKB49160.1	*Methanosarcina sp. Kolksee*	III	36
	WP_048167424.1	*Methanosarcina thermophila*	III	36
	WP_048123280.1	*Methanosarcina vacuolata*	III	36
*	ABE53176.1	*Methanococcoides burtonii* DSM 6242	IIIB	100
*	WP_048193098.1	*Methanococcoides methylutens*	IIIB	88
*	ADE36448.1	*Methanohalophilus mahii* DSM 5219	IIIB	85
*	WP_015052263.1	*Methanolobus psychrophilus*	IIIB	86
*	WP_023846593.1	*Methanolobus tindarius*	IIIB	88
*	WP_015325294.1	*Methanomethylovorans hollandica* (B)	IIIB	83
*	WP_013720421.1	*Methanosaeta concilii*	IIIB	77
*	AEH60135.1	*Methanosalsum zhilinae* DSM 4017	IIIB	84
	KNZ41810.1	*Acetobacterium bakii*	IV	27
	4NAS_A	*Alicyclobacillus acidocaldarius Subsp. acidocaldarius* DSM 446	IV	27
	EAS48331.1	*Aurantimonas manganoxydans* SI85-9A1	IV	28
	3FK4_A	*Bacillus cereus* ATCC 14579	IV	25
	AHY51744.1	*Bradyrhizobium japonicum* SEMIA 5079	IV	26
	3NWR_A	*Burkholderia fungorum*	IV	25

1YKW_A	*Chlorobium tepidum*	IV	28
ABE60559.1	*Chromohalobacter salexigens* DSM 3043	IV	25
CUX56898.1	*Clostridium sp.* C105KSO14	IV	26
2OEL_A	*Geobacillus kaustophilus*	IV	24
WP_041368253.1	*Nakamurella multipartita*	IV	27
ABE41999.1	*Polaromonas sp.* JS666	IV	25
ABQ77998.1	*Pseudomonas putida* F1	IV	26
WP_006599467.1	*Pseudoramibacter alactolyticus*	IV	26
WP_015049613.1	*Thermacetogenium phaeum*	IV	30

SUPPLEMENTAL FIGURE LEGENDS

SUPPLEMENTAL FIGURE S1. Structure-based sequence alignment of form II and form III Rubisco LSu sequences. Conserved residues are *boxed*, strictly conserved residues have a *red background*, residues well conserved within a group are indicated by *red letters*, and the remaining residues are in *black letters*. Gaps are represented by dots. Residue numbering along the top refers to MbR. *Symbols above* blocks of sequences correspond to the secondary structure of MbR: α, α-helix; β, β-strand; η, 3_{10}-helix. The secondary structure elements were named βA, βB..., αA, αB...etc, except for the strands and helices of the C-terminal βα barrel units, which were named β1, β2..., α1, α2, etc according to Knight *et al.,* 1990 (1). *Symbols below* blocks of sequences indicate the location of residues implicated in lock site formation (black triangles), the catalytic lysine residue (black star) and regions with consistent sequence-structure variation between the form II and form III lineages (*i-viii*, also see Fig. S2F). The position of the sequence coding catalytically-important loop 6 connecting β6 and α6 is indicated. The Rubisco assembly domain, αJ, is boxed and labelled in cyan. The sequence alignment was created using the PDB coordinates/UniProt sequences 5MAC/Q12TQ0 (MbR), 5RUB/P04718 (*R. rubrum*), 4LF1/Q6N0W9 (*R. palustris*), 5C2G/A0A0X1KHE5 (*Gallionaceae* sp.), 1GEH/O93627 (*T. kodakarensis*) and 2D69/O58677 (*P. horikoshii*).

SUPPLEMENTAL FIGURE S2. Structure comparison of MbR, form II and form III Rubiscos. Two views of the individual superposition of the LSu from (A) 5RUB, purple, (B) 4LF1, blue, (C) 52C2, cyan, (D) 1GEH, yellow and (E) 2D69, dark green onto MbR, light grey (RAD colored red). The length and structure of particular LSu loops are characteristic of either the form II or form III Rubisco lineages: superposition of these regions from all the analysed structures upon MbR are shown as inset boxes in (F). The MbR LSu folds like the form III (*i.e.* archaeal) Rubiscos in several of these loops: (i) the N-terminal loop between αB and βA folds as a helix in the proteobacterial structures; (iii) the loop between helices ηA and αE in the N-terminal domain is longer in the form II Rubiscos than in MbR or the form III Rubiscos; (*iv*) the loop before β1 in the C-terminal domain is longer in the form II enzymes than in MbR and the form III Rubiscos; (*viii*) MbR does not have the extended C-terminal domain common to form II, but not to form III Rubiscos. In other loop regions, the MbR LSu exhibits distinctly form II-like (*i.e.* proteobacterial-like) structure: (*ii*) the loop between strands βB and βC of the N-terminal domain is shorter in MbR and the form II Rubiscos than in the form III Rubiscos; (*v*) the C-terminal domain “bottom of the barrel" loop between helix α3 and strand β4 is shorter in form III Rubiscos than the corresponding loop in MbR and form II Rubiscos; (*vi*) αI-βE in loop 5 has distinct conformations characteristic of either the MbR and form II or form III structures; (*vii*) the structure from βF to βG is conserved between MbR and form II Rubiscos, with notable variation in the form III structures. C, the C-terminus of each LSu. Insets are not to scale and are numbered according to their ordering in primary sequence, and are similarly numbered in Figure S1. The relative locations of these loops are indicated by dashed boxes in (A).

SUPPLEMENTAL FIGURE S3. Detailed unrooted minimum evolution phylogenetic tree of Rubisco LSu sequences: a detailed view of the phylogenetic tree shown in *Fig. 6*. See *Fig. 6* figure legend for details. Grey arrows indicate Rubiscos from organisms in the *Methanosarcinales* Order that do not contain the assembly motif.

Supplemental Figure S1

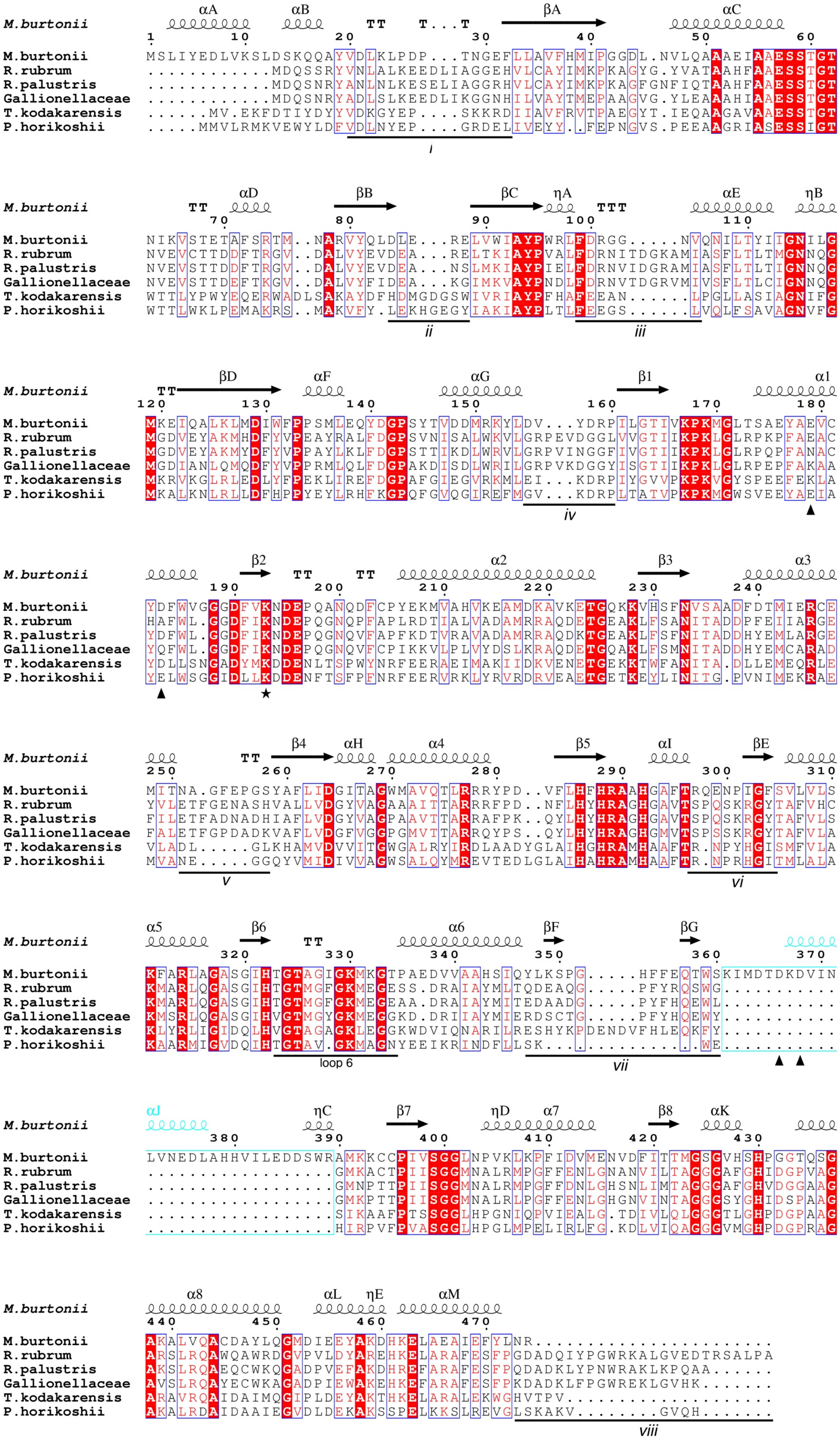

Supplemental Figure S2

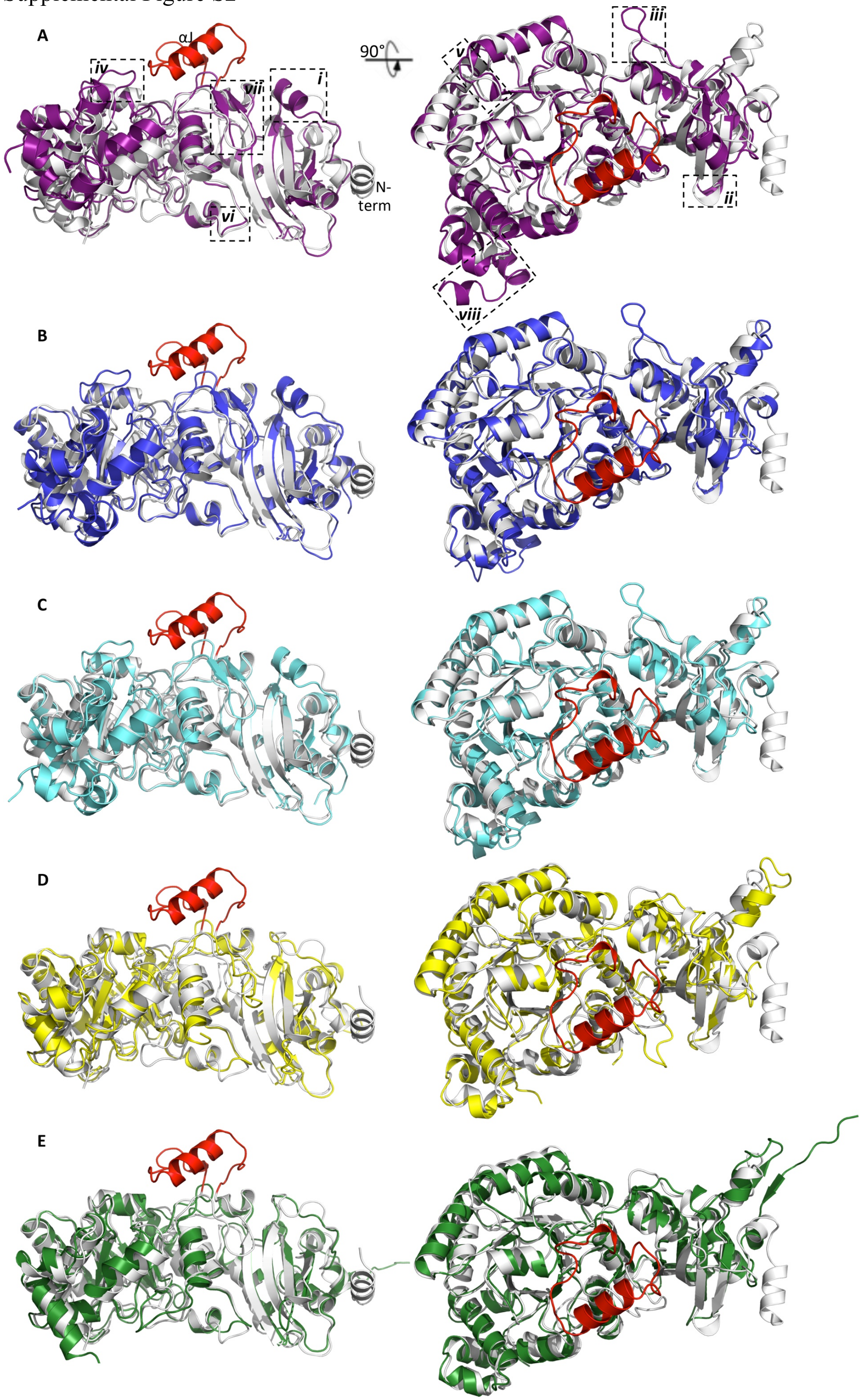

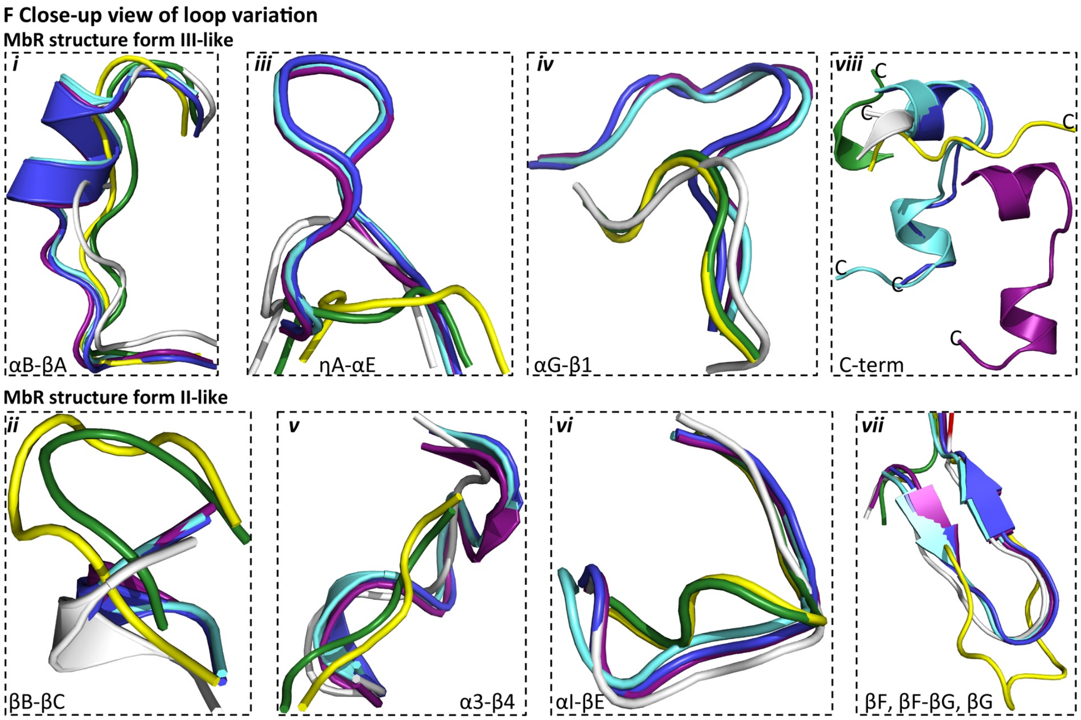
F Close-up view of loop variation
MbR structure form III-like
i
αB-βA
iii
ηA-αE
iv
αG-β1
viii
C
C
C
C
C
C
C-term
MbR structure form II-like
ii
βB-βC
v
α3-β4
vi
αI-βE
vii
βF, βF-βG, βG

Supplemental Figure S3

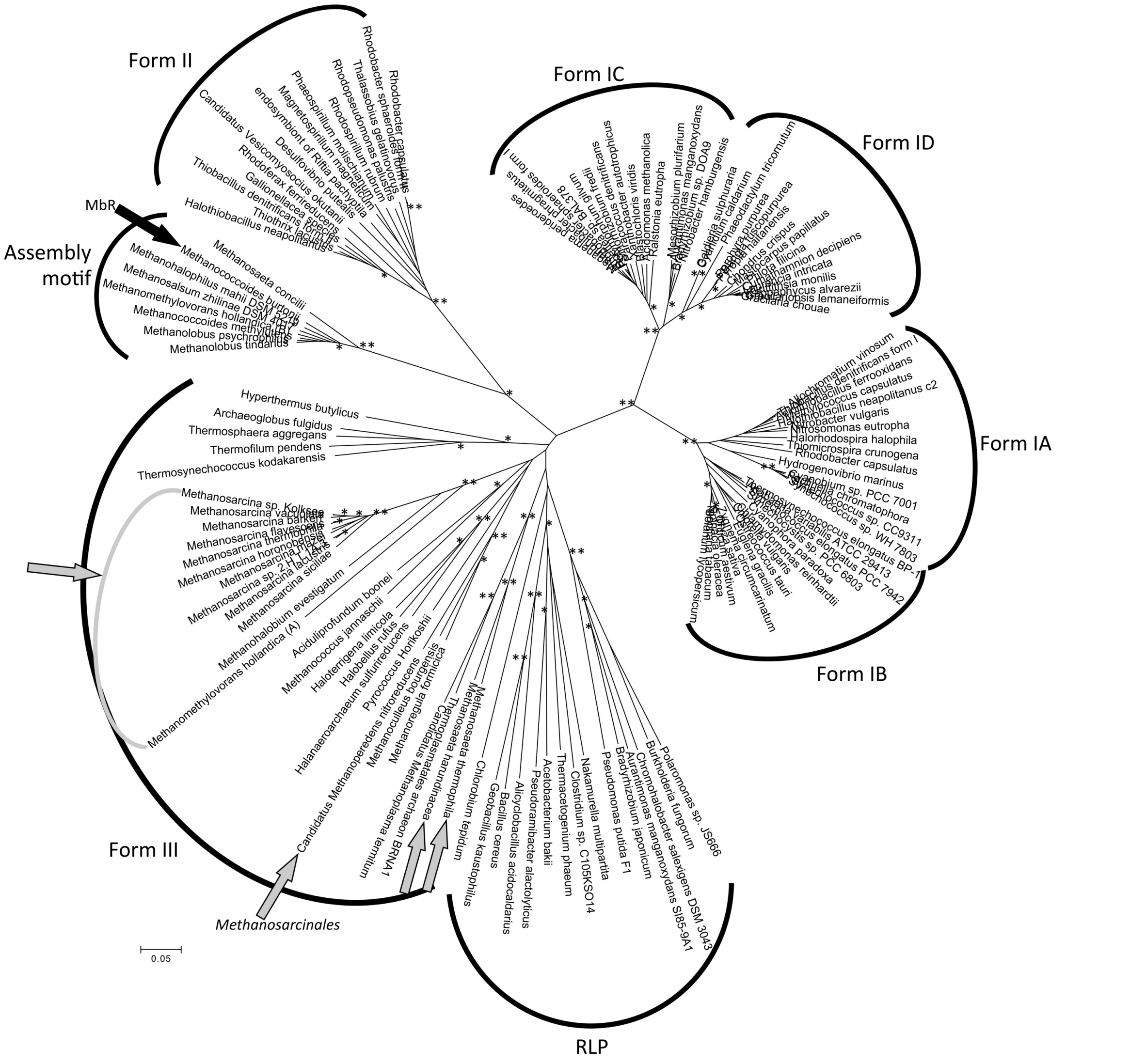

REFERENCES

1. Knight, S., Andersson, I., and Brändén, C. I. (1990) Crystallographic analysis of ribulose 1,5-bisphosphate carboxylase from spinach at 2.4 A resolution. Subunit interactions and active site. *J. Mol. Biol.* **215**, 113–160
